# Identification and temporal expression profiles of cuticular proteins in the endoparasitoid wasp, *Microplitis mediator*

**DOI:** 10.1111/1744-7917.12711

**Published:** 2019-08-06

**Authors:** Olga Volovych, Zhe Lin, Jie Du, Hong Jiang, Zhen Zou

**Affiliations:** ^1^ State Key Laboratory of Integrated Management of Pest Insects and Rodents Institute of Zoology, Chinese Academy of Sciences Beijing China; ^2^ University of Chinese Academy of Sciences Beijing China

**Keywords:** cuticular protein, expression pattern, *Microplitis mediator*, transcriptome

## Abstract

Recently, parasitoid wasp species *Microplitis mediator* has evoked increasing research attention due to its possible use in the control of Lepidoptera insects. Because insect development involves changes in cuticle composition, identification and expression analysis of *M. mediator* cuticular proteins may clarify the mechanisms involved in parasite development processes. We found 70 cuticular proteins from the *M. mediator* transcriptome and divided them into seven distinct families. Expression profiling indicated that most of these cuticular protein genes have expression peaks specific for one particular developmental stage of *M. mediator*. Eggs and pupae have the highest number of transcriptionally active cuticular protein genes (47 and 52 respectively). Only 12 of these genes maintained high expression activity during late larval development. Functional analysis of two larval proteins, MmCPR3 and MmCPR14, suggested their important role in the proper organization of the cuticle layers of larvae. During *M. mediator* larval development, normal cuticle formation can be supported by a limited number of cuticular proteins.

## Introduction

Insect cuticle is a complex multilayer structure that provides a physical barrier against external environmental factors. The procuticle is the thickest part of the cuticle, and it includes chitin fibrils embedded together with structural cuticular proteins (CPs) (Andersen *et al*., 1995). Composition of the CPs determines the physical properties of the cuticle such as elasticity and rigidness (Dittmer *et al*., 2012). To clarify the difference between CPs, annotated proteins of different insect species were divided into 13 families based on their chitin‐binding domain (ChtBD) or conserved motifs (Willis, 2010). The most numerous members of insect CPs form the CPR family, which possess the Rebers and Riddiford (RR) consensus region (Andersen *et al*., 1995). Based on sequence variations, this family can be divided into the subfamilies: RR‐1 and RR‐2 (Andersen, 2000). The distribution of RR‐1 and RR‐2 subfamilies correlates with the mechanical characteristics of the cuticle (soft and rigid types, respectively) (Gu & Willis, 2003; Zhou *et al*., 2016). The proteins from this family play important roles in adjusting the mechanical properties of the cuticle for specific body requirements. They provide body protection while maintaining flexibility. Another large group includes the CPs analogous to peritrophins (CPAP). This includes two families, CPAP1 and CPAP3 (Jasrapuria *et al*., 2010). The CPAP1 and CPAP3 families are characterized by containing respectively one or three of the six cysteine‐containing type‐2 ChtBD (ChtBD2). The proteins from these families are crucial for normal molting and development (Jasrapuria *et al*., 2012; Petkau *et al*., 2012; Pesch *et al*., 2015). The TWDL (Tweedle) family is widespread among insects and is characterized by a defined pattern with high proline density insertions. TWDL proteins are important for maintaining body shape during insect development (Guan *et al*., 2006). The CPF (CPs with a 42–44 amino acid motif) and CPFL (CPF‐like) families are also common in insects. Despite being conservative among insects, the functions and localizations of these families are poorly known. The chitin‐binding abilities of these CPs still require further investigation (Willis, 2010; Vannini *et al*., 2014). The CPLCA (CPs rich in alanine residues), CPLCG (CPs with conserved glycine residues), CPLCW (CPs with invariant tryptophan residue), and CPLCP (CPs rich in proline residues), are four families of low‐complexity CPs. They are characterized by a high density of specific amino acids or repeats (Cornman & Willis, 2009). Apidermin proteins consist of a family typical for Hymenoptera species and were annotated in *Apis mellifera*, *Nasonia vitripennis*, *Bombus terrestris*, and *Bombus impatiens* (Kucharski *et al*., 2007; Werren *et al*., 2010; Sadd *et al*., 2015; Micas *et al*., 2016). Proteins of this family are characterized by strong hydrophobicity due to their high alanine content.

Analysis of insect genomes showed that around 1% of protein‐coding genes are represented by CP (Ioannidou *et al*., 2014). CPs have been identified and analyzed on genomic, transcriptomic, or proteomic levels in more than 20 insect species (Shofuda *et al*., 1999; Adams *et al*., 2000; Consortium, 2006; He *et al*., 2007; Togawa *et al*., 2007; Futahashi *et al*., 2008; Cornman & Willis, 2009; Gallot *et al*., 2010; Liang *et al*., 2010; Werren *et al*., 2010; Carrasco *et al*., 2011; Suetsugu *et al*., 2013; Sadd *et al*., 2015; Micas *et al*., 2016; Jan *et al*., 2017; Yang *et al*., 2017; Zhao *et al*., 2017; Zhou *et al*., 2017; Pan *et al*., 2018). However, research related to CPs has mostly focused on Diptera and Lepidoptera. The number of annotated CPs is highly variable among insects, ranging from 63 in *Pediculus humanus corporis* to 305 in *Aedes aegypti* (Ioannidou *et al*., 2014). The differences could be related to the variability of the body size and habitats. The heterogeneity of specific features for classification is also high. Some families are characterized by the type of ChtBD, and others are classified based on possession of specific amino acid repeats.

*Microplitis mediator* (Haliday) (Hymenoptera: Braconidae) is an endoparasitoid wasp, which parasitizes larvae of Lepidoptera species such as *Helicoverpa armigera*, *Mamestra brassicae*, and *Heliothis virescens* (Lauro *et al*., 2005; Li *et al*., 2006). To facilitate the larval development of the parasitoid wasp inside the host body, different strategies are used to disrupt the metabolic, immune, and development processes of the host. Similar mechanisms occur during viral and fungal infection in *H. armigera*. These involve the manipulation of host processes for infection propagation (Xiong *et al*., 2015; Yuan *et al*., 2017). The composition of structural CPs is variable during insect development and strongly associated with the molting process (Schwartz & Truman, 1983; Charles, 2010; Soares *et al*., 2011).

The main goal of this study was to determine features in CP composition and expression patterns that are crucial for the development of parasitoid larvae and successful pupation.

## Materials and methods

### Experimental insects

The colony of *M. mediator* was reared in the laboratory at 26 ± 1 °C with a 14 : 10 h (L : D) photoperiod and 60% ± 10% relative humidity as previously described (Lin *et al*., 2018). *H*. *armigera* larvae were reared on an artificial diet at 28 ± 1 °C, 60% ± 10% relative humidity, and a 14 : 10 h (L : D) photoperiod (Xiong *et al*., 2015). *M. mediator* parasitizes the 2nd instar *H*. *armigera* larvae and produces a single offspring per host. The parasitized *H. armigera* larvae were reared individually in containers.

### CP genes annotation and phylogenetic analysis

Two strategies were followed to identify putative *M. mediator* CPs: high similarity to known CP sequences and possession of specific domains or amino acid motifs that characterize the CP families (Futahashi *et al*., 2008). The cut‐off criterion of amino acids motifs was 50% similarity. In the first annotation step, the sequences for known CPs were collected from seven insect species: *A. aegypti* (Cornman & Willis, 2009), *A. mellifera*, *N. vitripennis* (cuticle DB bioinformatics.biol.uoa.gr/cuticle DB; Ioannidou *et al*., 2014), *Drosophila melanogaster* (Cornman, 2009), *Manduca sexta* (Dittmer *et al*., 2015), *Bombyx mori* (Futahashi *et al*., 2008), and *Tribolium castaneum* (Jasrapuria *et al*., 2010). Known sequences, divided by families, were used as queries for Basic Local Alignment Search Tool (BLAST) search against the *M. mediator* transcriptome. For division into different CP families, all putative sequences were checked for the possession of specific domains using National Center for Biotechnology Information (NCBI) Batch CD‐Search (Marchler‐Bauer *et al*., 2015) and PATTINPROT tools. Results were verified with the CutProtFam prediction tool (Ioannidou *et al*., 2014).

The CPR family was identified by the possession of the RR consensus as chitin_bind_4 (ChtBD4) (Pfam, PF00379). Division of the family into RR‐1 and RR‐2 subfamilies was accomplished according to specific amino acid motif possession. The signature motif of RR‐1 consensus is GxFxYxxPDGxxxxVxYxADENGYQPxGAHLP. Consensus of RR‐2 subfamily motif is xEYDxxPxYxFxYxVxDxHTGDxKSQx ExRDGDVVxGxYSLxExDGxxRTVxYTADxxNGFNAVxxEx (Iconomidou *et al*., 2005; Willis *et al*., 2005). Further division of sequences was completed using the CuticleDB tool based on profile hidden Markov models (Karouzou *et al*., 2007). We also tried to annotate proteins with another type of modified RR consensus, called RR‐3 proteins. The specific features of RR‐3 proteins have not been generalized until now, so we used previously annotated proteins as a query for annotation (Table S4).

CPAP1 and CPAP3 families were identified by possessing specific Chitin binding Peritrophin‐A domain (ChtBD2 Pfam, PF01607; CBM_14, pfam01607) revealed by NCBI Batch CD‐Search. CPAP1 family has one ChtBD, and CPAP3 possesses three ChtBDs. To annotate TWDL family proteins, we used the amino acid pattern: YVLX_20‐23_KPEVyFiKY(R/K)t (Guan *et al*., 2006). Members of the CPCFC family were searched using the YPAGVNPAACPNYPYCD pattern. Proteins of the CPF family were searched using the following motif: VSxYSKAVDTPFSSVRKxDxRIVNxA (Togawa *et al*., 2007). CPFL family members were identified by the motif: LxYSAAPAVSHVAYxGxGxxYGW (Togawa *et al*., 2007). The CPLCP family was characterized by high frequency of proline residues that usually appear as PV and PY amino acid pairs. A characteristic of the Apidermin family is high content of alanine residues, which are represented in a form of tetra‐peptide AAPA/V. Full sequences of CPAP1 and CPAP3 proteins were used to perform phylogenetic analysis. The sequence of ChtBDs of CPAP1 and CPAP3 was obtained after NCBI Batch CD‐Search (Marchler‐Bauer *et al*., 2015). ChtBD sequences of CPAP1 and CPAP3 proteins were aligned using Clustal Omega (Sievers *et al*., 2011) and used in phylogenetic and conservative pattern analysis. For CPR proteins, full RR consensus was used for alignment and further logo generation and phylogenetic analysis. WebLogo 3 was used to visualize conservative amino acids of ChtBDs (CPAP1, CPAP3) and RR consensuses (RR‐1, RR‐2) (Crooks *et al*., 2004). Phylogenetic trees were constructed using the neighbor‐joining method (MEGA 7.0.18), with 1500 bootstrap replications. Accession numbers of CPs used for phylogenetic analysis are presented in Table S4. The accession numbers for CPAP1 and CPAP3 analysis were obtained from Tetreau *et al*. (2015).

### CP gene expression profiling

The life cycle of *M. mediator* has four stages: egg, larva, pupa, and adult. Six RNA‐seq libraries corresponding to different developmental stage samples were prepared. Host larvae were dissected on the first day after parasitization for the collection of *M. mediator* eggs (*n* = 400), on the second day after parasitization for 1st instar larvae (*n* = 200), on the fifth day after parasitization for 2nd instar larvae (*n* = 20), and on the seventh day after parasitization for 3rd instar larvae (*n* = 20), following a previous study (Wang *et al*., 2017). The pupal stage (*n* = 15) and adult (*n* = 10) females were also collected. Another adult sample (females *n* = 5 and males *n* = 5) was collected for relative expression quantification. The samples were washed twice in phosphate‐buffered saline (PBS) and homogenized in Trizol reagent.

To generate six libraries, total RNA was extracted using an RNeasy mini kit (Qiagen, Germantown, MD, USA). RNA integrity was checked using an Agilent 2100 Bioanalyzer (Agilent Technologies, Palo Alto, CA, USA). Ten micrograms of RNA from each sample were used to isolate messenger RNA (mRNA) through oligo (dT) magnetic beads (Invitrogen, Carlsbad, CA, USA). The 2 × 100 bp paired‐end RNA‐seq libraries were carried out using Illumina Truseq RNA Sample Preparation kit V2 and sequenced using the Illumina HiSeq™ 2000 platform (Illumina, San Diego, CA, USA). The sequence data were processed as shown in a previous paper (Lin *et al*., 2019).

Library reads were matched to specific transcripts using RSEM software (Grabherr *et al*., 2011; Li & Dewey, 2011). Changes in CP gene expression were calculated by the number of reads that mapped to the genes and are represented as fragments per kb per million mapped reads (FPKM). FPKM numbers for each CP gene are shown in Table S1. Heatmap 2 suite in R was used to visualize hierarchical clustering of calculated FPKM values (Warnes *et al*., 2015). Pearson correlation‐based metric and the average linkage clustering method were set as clustering parameters.

### Quantitative polymerase chain reaction (qPCR)

Primers for qPCR analysis were designed by Primer3 online (Rozen & Skaletsky, 2000) and checked using OligoAnalyzer 3.1. For qPCR reactions SuperReal PreMix Plus (SYBR Green) (Tiangen, BJ, China) was used according to the manufacturer suggestions. Thermal conditions of reactions in the MX3000P machine (Stratagene, La Jolla, CA, USA) were 95 °C, 2 min; (95 °C, 20 s; 59 °C, 20 s; 68 °C, 20 s) × 40 cycles; 95 °C, 1 min; 59 °C, 30 s; 95 °C, 30 s. Reactions were replicated at least thrice.

Several house‐keeping genes were verified as appropriate reference genes for data normalization in *A. mellifera* studies. These included ribosomal proteins Amrp49 and the element of cytoskeleton β‐actin (Iovchev *et al*., 2006; Scharlaken *et al*., 2008; Soares *et al*., 2011). To choose an appropriate reference gene for qPCR results normalization, we checked the fluctuation of FPKM number changes of these two candidate genes through the entire transcriptome. The β‐actin gene had low fluctuation of FPKM numbers during developmental stages of *M. mediator*, and it was chosen as the gene for qPCR normalization. The primers for CPs and β‐actin are shown in Table S2.

The qPCR data were analyzed by the 2^−ΔΔ^ *^C^* ^t^ calculation method (Livak & Schmittgen, 2001). The adult stage has the longest duration in the *M. mediator* life cycle with low fluctuation of CP genes expression from the first day of adult eclosion until the old adult. So, *M. mediator* adult samples were therefore treated as the calibrator for data analysis. GraphPad Prism 6 was used for statistical analysis and data visualization. One‐way analysis of variance (ANOVA) and Uncorrected Fisher's Least Significant Difference were used for multiple comparisons. A probability of *P* < 0.05 was accepted as statistically significant.

### RNA interference (RNAi)

Double‐stranded RNA (dsRNA) for *MmCPR14* and *MmCPR3* was prepared using the T7 RiboMAX Express RNAi system Kit (Promega, Madison, WI, USA) according to the manufacturer protocol. The primers used to amplify the template are presented in Table S2. The green fluorescent protein (GFP) sequence was used as a template to synthesize control dsRNA. On the fourth day after parasitization ice‐anesthetized *H. armigera* was injected into abdominal segment 10 using a nanoliter 2000 injector (World Precision Instruments, Sarasota, FL, USA). Each insect was injected with 100‒130 nL of dsRNA depending on its concentration (approximately 160 ng per insect). Injection of dsRNA was performed in slow speed mode (23 nL/s). Larvae were divided into three groups (*n* = 25 each) based on the type of injected dsRNA (dsGFP, dsMmCPR3, or dsMmCPR14). Non‐injected samples of *M. mediator* larvae were added as an additional control for cuticle thickness measurement.

Non‐parasitized *H. armigera* (*n* = 25 each treatment group) were also injected with dsMmCPR3, dsMmCPR14 and dsGFP as a control to exclude the possibility of co‐interference of dsRNA with *H. armigera* CP genes. The number of surviving larvae was recorded on the first, second, third and fourth day post‐injection. Kaplan–Meier survival analysis was used to compare survival rates of the different treatments (Kaplan & Meier, 1958). To analyze the knock‐down efficiency of dsRNA injection, complementary DNAs (cDNAs) were synthesized from total RNA samples isolated from the whole body of *M. mediator* larvae (*n* = 4 for each injected group) on the third day post‐injection for further qPCR.

### Cuticle thickness evaluation

On the third day post‐injection, six randomly selected *M. mediator* for each injected group were dissected and evaluated using histological analysis. In order to prepare samples for cryosectioning *M. mediator* larvae were rinsed with PBS and fixed with 4% paraformaldehyde overnight at 4 °C. Fixed samples were soaked with 30% sucrose as a cryoprotector and then frozen in tissue‐freezing medium. The whole body of *M. mediator* larvae was used for preparing sagittal cross‐sections with 7 *µ*m thickness using cryomicrotome Leica CM1950 (Leica, Wetzlar, Germany). To estimate cuticle thickness, a series of sections were stained with chitin‐binding dye Calcofluor White M2R (Sigma‐Aldrich, St. Louis, MO, USA) and visualized using a LSM 780 Confocal Microscope (Zeiss, Dublin, CA, USA) (440 nm excitation wavelength and 500 nm wavelength emission parameters). We did not observe a statistically significant difference between the dorsal and ventral locations of the cuticle in *M. mediator* larvae. So, for further analysis, we randomly selected cuticle regions with the exceptions of the head and the last posterior abdominal segment. Digital images of six selected areas for every sample were recorded and used for manual morphometric measurements with ImageJ. All measurement sets were tested for normality using D'Agostino‐Pearson omnibus normality test and then compared with the Mann–Whitney test.

### Data availability

All sequence data and the final *M*. *mediator* transcriptome described in this study are available under the BioProject accession number PRJNA524252. Illumina sequence reads have been deposited in the NCBI SRA database under the following accession numbers (Egg: BioSample SAMN11019679, Reads SRR8648170; 1st instar larvae: BioSample SAMN11019681, Reads SRR8648171; 2nd instar larvae: BioSample SAMN11019687, Reads SRR8648168; 3rd larvae: BioSample SAMN11019689, Reads SRR8648169; pupae: BioSample SAMN11019696, Reads SRR8648166; adult: BioSample SAMN11019697, Reads SRR8648167). Unigenes of *M*. *mediator* are available under the NCBI Transcriptome Shotgun Assembly accession numbers associated with the same BioProject. Accession numbers and amino acid sequences of *M. mediator* annotated CPs are presented in Table S3. The sequences were deposited in the GenBank under accession numbers from MK632949 to MK633018.

## Results

### CP gene transcriptome‐wide annotation

Analysis of the six *M. mediator* transcriptomic libraries identified 70 putative CP gene transcripts. Among these, 54 were obtained with full‐length sequences. Others were partially annotated without C‐ or N‐terminals (3 and 13 CPs respectively) (Table S1). Based on amino acid sequence analysis and domain identification, CP transcripts were divided into seven distinct families (CPR, CPAP1, CPAP3, TWDL, CPLCP, CPF, and Apidermin).

### CPR genes

**Figure 1:**
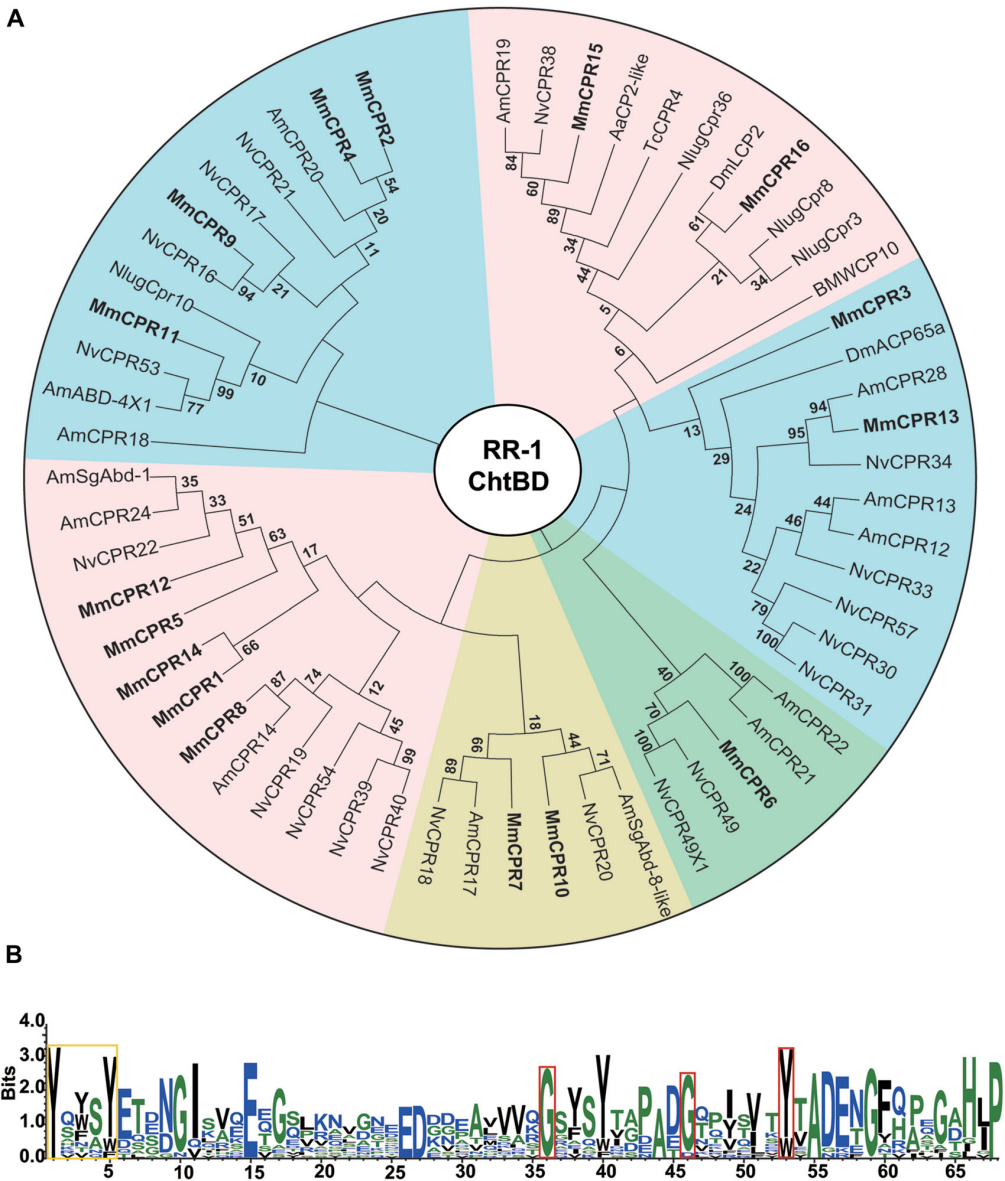
Rebers and Riddiford (RR)‐1 proteins phylogenetic analysis and amino acid pattern of RR consensus. (A) RR‐1 subfamily phylogenetic relationships among *Microplitis mediator* (Mm), *Apis mellifera* (Am), *Nasonia vitripennis* (Nv), and several genes from functional studies of *Tribolium castaneum* (Tc), *Aedes albopictus* (Aa), *Bombyx mori* (BM), *Nilaparvata lugens* (Nlug), and *Drosophila melanogaster* (Dm). Full RR consensuses were used for multiple sequence alignment. RR‐1 proteins from *M. mediator* are indicated in bold. (B) Pattern was generated on RR consensus of 16 RR‐1 proteins. It consists of 68 amino acids and contains N‐terminal aromatic triad pattern (marked in yellow frame) and conservative glycine and tyrosine residues in the specific pattern G‐x(8)‐G‐x(6)‐Y (marked with red frames). It was generated by WebLogo 3 (Crooks *et al.*, 2004), based on Clustal X alignment.

CPR was the most abundant family annotated in *M. mediator*, with 40 protein genes annotated. Among these, there were 16 RR‐1, 23 RR‐2, and 1 RR‐3 proteins annotated in this study. We used RR consensus sequences of *M. mediator*, *A. mellifera*, and *N. vitripennis* together with other CP RR from different functional studies about the RR‐1 subfamily (Gallot *et al*., 2010; Poelchau *et al*., 2013; Noh *et al*., 2014; Jan *et al*., 2017; Pan *et al*., 2018) to further analyze the phylogenetic grouping of annotated proteins. Accession numbers are presented (Table S4). After deleting non‐specific groups with low bootstrap values, we obtained the phylogenetic tree for further analysis (Fig. 1A). Several CPs, such as MmCPR1, 3, and 14 had no clear orthologs among the represented sequences, and formed separate branches. Therefore, we believe that these CPs are species‐specific. MmCPR6 and 9 are possible orthologs with *N. vitripennis* NvCPR49 and NvCPR16, respectively. These CPs could be conservative among parasitoid wasp species. Several RR‐1 members of *M. mediator* (MmCPR7, 8, 11, and 13) were grouped with *N. vitripennis* as well as with *A. mellifera* CPs under high bootstrap values. It is possible that these are also conservative among Hymenoptera species.

**Figure 2:**
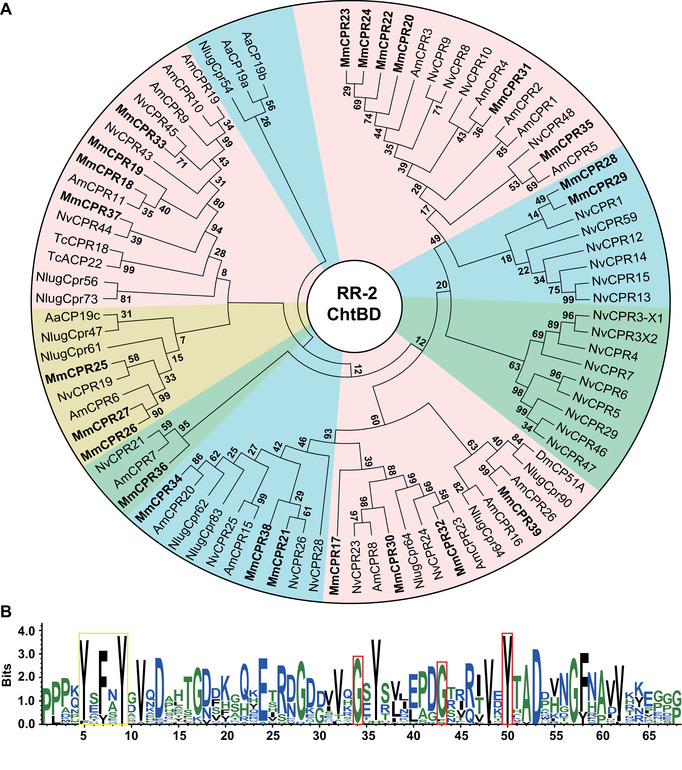
Rebers and Riddiford (RR) proteins phylogenetic analysis and amino acid pattern of RR consensus. (A) RR‐2 subfamily phylogenetic relationships among *Microplitis mediator* (Mm), *Apis mellifera* (Am), *Nasonia vitripennis* (Nv) *Tribolium castaneum* (Tc), *Aedes albopictus* (Aa), *Nilaparvata lugens* (Nlug) and *Drosophila melanogaster* (Dm). Full RR consensuses were used for multiple sequence alignment. RR‐2 proteins from *M. mediator* are indicated in bold. (B) Pattern was generated on RR consensus of 23 RR‐2 proteins. It consists of 68 amino acids and contains N‐terminal aromatic triad in the specific pattern Y‐x‐Y/F‐x‐Y (marked in yellow frame), and conservative glycine and tyrosine residues in the specific pattern G‐x(8)‐G‐x(6)‐Y (marked with red frames). Logo was generated by WebLogo 3, based on Clustal X alignment.

A similar phylogenetic tree was constructed using RR‐2 consensus of *M. mediator* together with the other species (Fig. 2A). Several CPs MmCPR20, 22, 23, and 24 were separate from other CPs and had no clear orthologs among the presented sequences. It is possible that these RR‐2 proteins are species‐specific for *M. mediator*. MmCPR32 and *N. vitripennis* NvCPR24 shared a common branch with AmCPR23 with high bootstrap value. Similarly, MmCPR33 has a common branch with NvCPR45, suggesting that these two proteins are possibly conservative to parasitoid wasp species. MmCPR26 and 27, which are analogous to AmCPR6, are the result of gene duplication. Several other proteins such as MmCPR34 and 39 are possible orthologs with *A. mellifera* AmCPR20 and 26, respectively. Two proteins MmCPR30 and 36 grouped with *N. vitripennis* and *A. mellifera* CPs by a common branch with high bootstrap values. They are possibly conservative among Hymenoptera species. Despite higher representation of annotated RR‐2 proteins in *M. mediator*, the number of possible orthologs with other Hymenoptera species was relatively low.

Analysis of *M. mediator* RR consensus of RR‐1 and RR‐2 revealed typical consensus (Willis, 2010). *M. mediator* RR‐1 consensus consists of 68 amino acids, with a conservative pattern G‐x(8)‐G‐x(6)‐Y (Fig. 1B). MmCPR3 has slightly increased the pattern to G‐x(9)‐G‐x(6)‐Y with the insertion of additional alanine. In MmCPR6, glycine on the second position of the pattern is substituted by aspartic acid. Aromatic amino acid tyrosine is substituted by tryptophan in three RR‐1 proteins: MmCPR7, 8, and 10 on the third position of the pattern. An aromatic triad in the N‐terminal (Willis, 2010) is typical for the CPR family, and it is usually represented by the motif Y‐x‐Y‐x‐Y. The original motif was found in four sequences MmCPR1, 3, 12, and 14. Proteins such as MmCPR6, 10, 15, and 16 lacked the classical aromatic triad or dyad. Instead, they possessed a single residue of tyrosine or tryptophan. In five sequences, the second tyrosine is substituted with another aromatic amino acid such as tryptophan (MmCPR9, 4, 2) or phenylalanine (MmCPR5 and 13). A second tyrosine of consensus is substituted with other amino acids (valine, asparagine, or threonine) in two sequences MmCPR7 and 11, which modify their consensus to an aromatic dyad. *M. mediator* RR‐2 consensus consists of 68 amino acids (Fig. 2B) that form a conservative pattern. The aromatic triad in the N‐terminal has the classical structure Y‐x‐Y/F‐x‐Y. The first position of the triad is highly conservative. The amino acid substitution appeared only in MmCPR32 with changes from tyrosine to tryptophan. The third position of the consensus has the same substitution in 20 proteins from 23 RR‐2. The fifth position of the consensus is a tyrosine residue for all RR‐2 CPs.

In the *M. mediator* transcriptome, RR‐3 proteins are represented by one putative protein, MmCPR40. It has a domain architecture similar to previously annotated RR‐3 proteins in *N. vitripennis* (NvitCPR58) and *M. sexta* (MsCPR149) (Willis, 2010; Dittmer *et al*., 2012). The common feature between MmCPR40, NvitCPR58, and MsCPR149 is the possession of a doubled RR consensus. RR‐3 protein frequently possesses a specific motif that consists of 18 amino acids in the conservative pattern (P/V)xDTPEVAAA(K/R)AA(H/F)‐xAA(H/Y) (Andersen, 2000). MmCPR40 possesses such a motif with 69% similarity.

### CPAP genes

**Figure 3:**
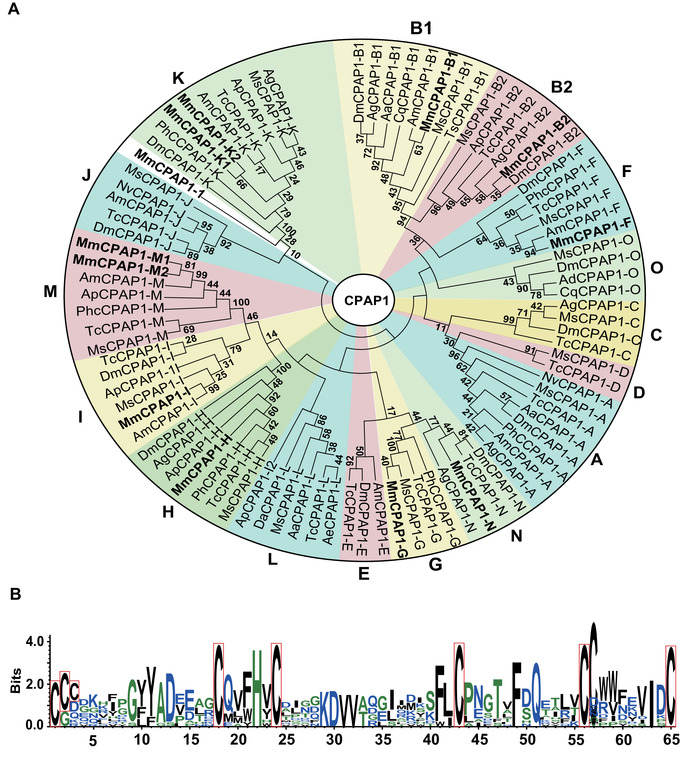
Cuticular proteins analogous to peritrophin 1 (CPAP1) proteins phylogenetic analysis and chitin‐binding domains (ChtBDs) amino acid pattern. (A) CPAP1 family phylogenetic relationships among 14 different species: *Acromyrmex echinatior* (Ae), *Acyrthosiphon pisum* (Ap), *Aedes aegypti* (Aa) *Anopheles darlingi* (Ad), *Anopheles gambiae* (Ag), *Apis mellifera* (Am), *Culex quinquefasciatus* (Cq), *Drosophila ananassae* (Da), *D. melanogaster* (Dm), *Manduca sexta* (Ms), *Microplitis mediator* (Mm), *Nasonia vitripennis* (Np), *Pediculus humanus corporis* (Phc), and *Tribolium castaneum* (Tc). Full amino acid sequences were used for multiple sequence alignment. CPAP1s from *M. mediator* are indicated in bold. (B) Consensus based on ChtBD sequences of 12 CPAP1 proteins. It consists of 65 amino acids and includes the consensus of six conservative cysteine residues in CX_14‐16_CX_5_CX_10‐18_CX_12_CX_7‐8_C pattern. Conservative cysteines are marked with a red frame.

**Figure 4:**
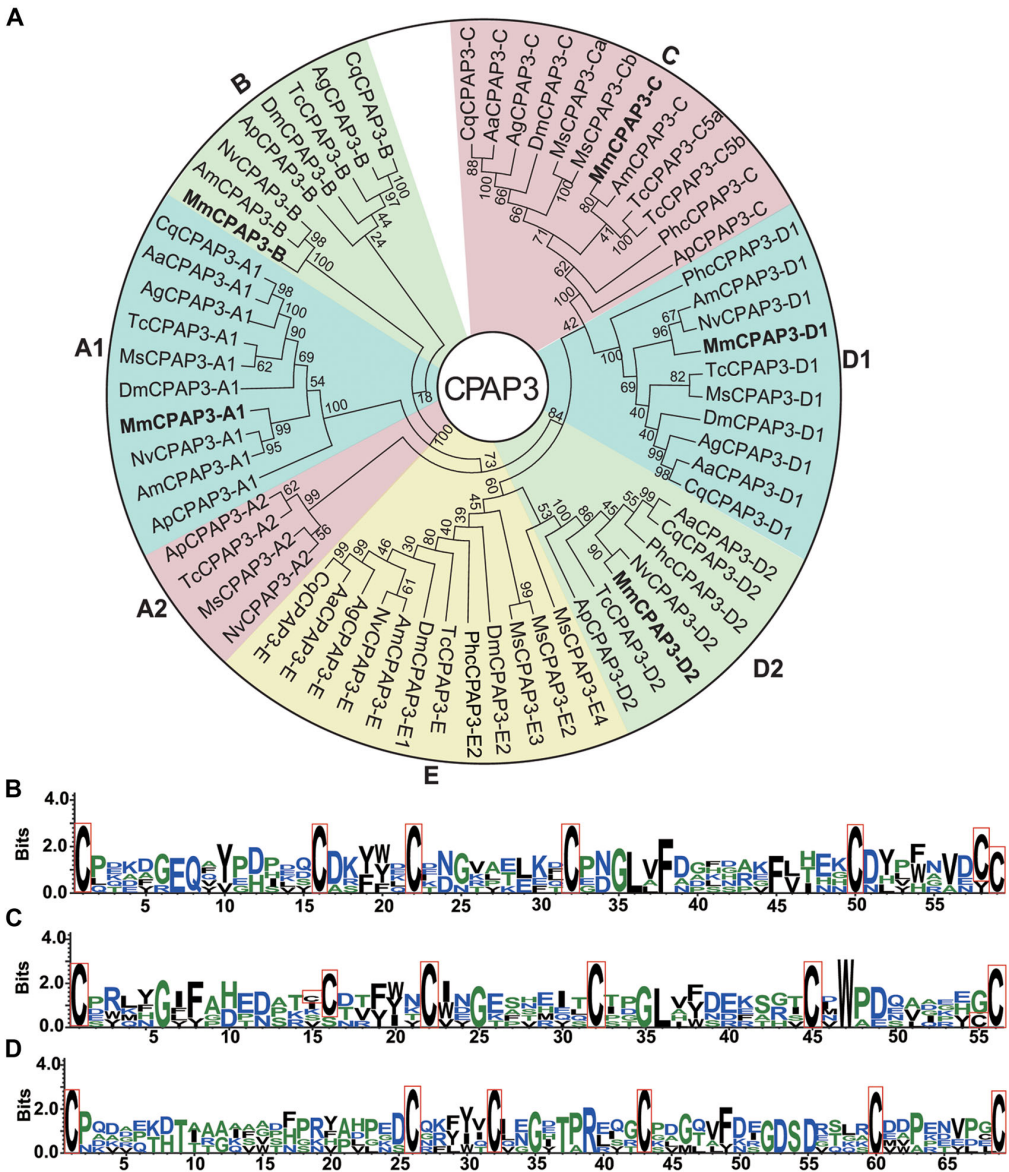
Cuticular proteins analogous to peritrophin 3 (CPAP3) proteins phylogenetic analysis and chitin‐binding domains (ChtBDs) amino acid patterns. (A) CPAP3 family phylogenetic relationships were generalized using full‐length sequences of 11 insect species *Acyrthosiphon pisum* (Ap), *Aedes aegypti* (Aa), *Anopheles gambiae* (Ag), *Apis mellifera* (Am), *Culex quinquefasciatus* (Cq), *D. melanogaster* (Dm), *Manduca sexta* (Ms), *Microplitis mediator* (Mm), *Nasonia vitripennis* (Np), *Pediculus humanus corporis* (Phc), and *Tribolium castaneum* (Tc). CPAP3 CPs of *M. mediator* are indicated in bold. Full amino acid sequences were used for multiple sequence alignment. (B) Consensuses based on ChtBD sequences of five proteins from the CPAP3 family. First, second, and third domains were analyzed and visualized separately. Consensus of the first domain includes 59 amino acids with six conservative cysteine residues in CX_12‐14_CX_5_CX_9_CX_13‐15_CX_7‐8_C pattern. (C) Consensus of second domain includes 56 amino acids with six conservative cysteine residues in CX_12‐14_CX_5‐6_CX_9_CX_12_CX_8‐10_C pattern. (D) Consensus of third domain includes 69 amino acids with six conservative cysteine residues in CX_11‐24_CX_5_CX_10‐11_CX_12‐16_CX_7‐8_C pattern. Conservative cysteines are marked with a red frame.

Transcriptome analysis revealed 17 genes from the CPAP group. They were divided into two families (12 CPAP1 and 5 CPAP3) according to the number of ChtBDs. We combined CPAP1 ChtBDs obtained from 13 insect species in previous studies (Tetreau *et al*., 2015) with sequences of *M. mediator* to conduct a phylogenetic analysis (Fig. 3A). Annotated CPAP1 proteins were divided among nine groups. The K and M groups possess two proteins, and the other groups contain one or no proteins. CPAP1‐1 has a low bootstrap number with other proteins from the K group, so its group classification requires further study. Phylogenetic analysis of CPAP3 was generalized using full‐length sequences from ten insect species (Tetreau *et al*., 2015). Accession numbers are presented in Table S4. Five annotated CPAP3 of *M. mediator* sort into five distinct clades: C, D1, D2, A1, and B with a single representative of each class (Fig. 4A).

The conservative structure of ChtBD2 of CPAP1 can be described with the following motif: CX_11‐24_CX_5_CX_9‐14_CX_12‐16_CX_6‐8_C (Jasrapuria *et al*., 2010). The position of six conservative cysteine residues is slightly different among different insect species. Based on the alignment of ChtBDs2 in *M. mediator*, a conserved motif was generated: CX_14‐16_CX_5_CX_10‐18_CX_12_CX_7‐8_C (Fig. 3B). Two proteins MmCPAP1‐K1 and MmCPAP1‐K2 had the longest distance between the first and second cysteine that consisted of 16 amino acids. The consensus for *M. mediator* annotated CPAP1 is similar to the accepted motif. The amino acid insert length between the first and second cysteine is less variable among ten proteins; the length difference consists of two amino acids. However, this did not affect the basic model. Proteins of the CPAP3 family had three ChtBD2 (Fig. 4 B–D). The domain pattern for the first ChtBD2 was: CX_12‐14_CX_5_CX_9_CX_13‐15_CX_7‐8_C (Fig. 4B). Highly conserved amino acid insets were located between the second and the third, the third and the fourth cysteine residues. The other two patterns were CX_12‐14_CX_5‐6_CX_9_CX_12_CX_8‐10_C and CX_11‐24_CX_10‐11_CX_12‐16_CX_7‐8_C (Fig. 4C and D). The third pattern was the longest, based on residue number range between first and second cysteine residues up to 24 amino acids. Considering the motif of all the domains we found that the annotated *M. mediator* CPAP CPs follow the structure of the classical motif.

### Other families of CP genes

The remaining families of CP are represented by a much smaller number of proteins among the total number of annotated CPs. However, some of these proteins are known to be important for normal insect development (Willis, 2010). Transcriptome analysis revealed four families in *M. mediator*. Two TWDL proteins were annotated in *M. mediator*. A characteristic of the TWDL family is the possession of domain DUF243, which consists of four conservative amino acid blocks. The CPF family of CPs is characterized by possessing 44 amino acids consensus. The CPFL family does not possess the consensus but has a high similarity with CPF protein on the C‐terminal (Willis, 2010). A single CPF protein was annotated in the *M. mediator* transcriptome. Transcripts from the CPFL family were not found in this study.

Based on BLAST search and amino acid composition analysis, we identified seven proteins from the CPLCP family. The main feature of this family is a high proline content that is represented in the form of amino acid pairs PV and PY (Cornman & Willis, 2009). The Apidermin family of CPs is a distinct feature of Hymenoptera species with annotated CP composition. Proteins of this family are characterized by a high frequency of alanine residues, and therefore, they are highly hydrophobic. The transcriptomic data of *M. mediator* showed three proteins from the Apidermin family with an alanine‐residue percentage of around 18%. Proteins from the CPCFC family were not identified in this study. Families such as CPLCG, CPLCA, and CPLCW previously were not annotated in Hymenoptera, so this study did not reveal any new features of *M. mediator* CPs variety, compared to other Hymenoptera species.

### Stage‐specific expression of CP genes

**Figure 5:**
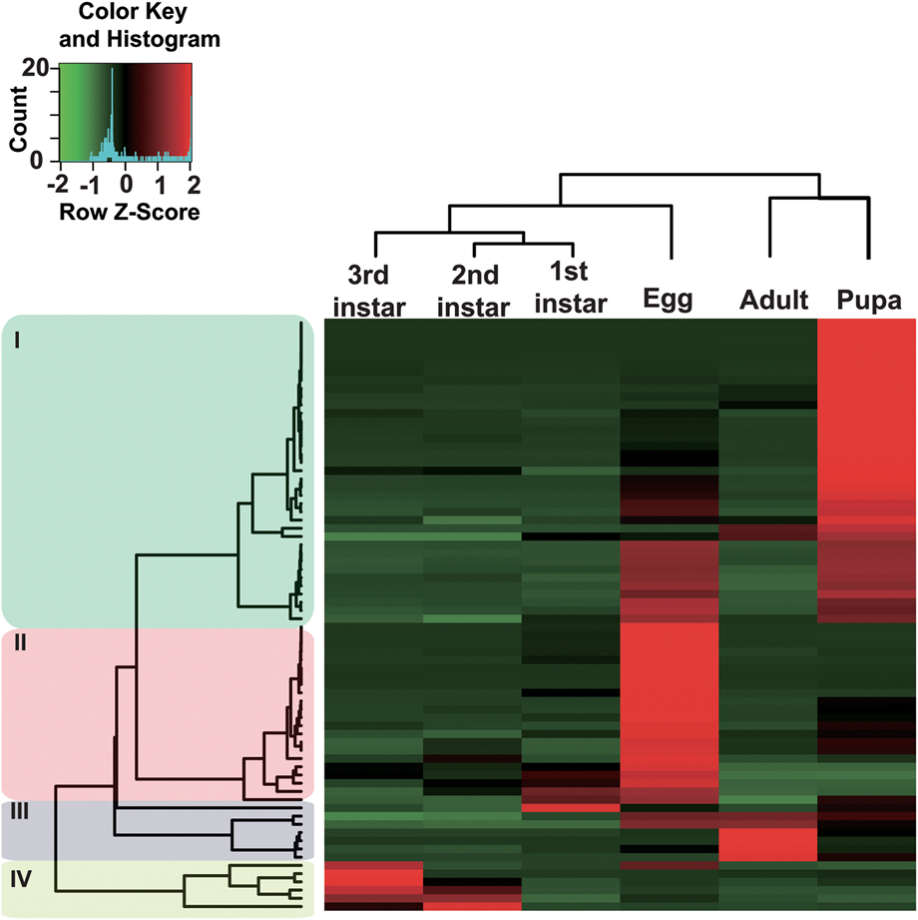
Hierarchical clustering analysis of the CP genes in *Microplitis mediator* during development. Fragments of kilobase per transcript per million fragment mapped (FPKM) values of 70 CPs genes among six different libraries (egg, 1st, 2nd, 3rd instar larva, pupa, and adult female) were analyzed to generate expression profiling. Changes in CPs genes expression were calculated by the number of reads that mapped to the genes and are represented in the form of FPKM values (Table S1). Hierarchical clustering of FPKM value was performed with the Pearson correlation‐based metric and average linkage clustering method. The heatmap is divided into four discrete clusters (Clusters I‐IV) and color coded on the left.

To visualize expression patterns of CPs during different *M. mediator* developmental stages, six RNA‐seq libraries from egg to adult stages were analyzed. Estimation of transcription expression levels was based on FPKM calculation. FPKM values of *M. mediator* CP genes are presented in Table S1. The hierarchical analysis divided development into four stage‐specific clusters: I (pupa), II (еgg), III (adult), and IV (larva) (Fig. 5). Two developmental points on the egg and pupal stages have the highest numbers of expressed CP genes compared to the other stages. The majority of CPs had expression peaks limited to one particular stage and formed four heat map clusters demonstrating their stage specificity during *M. mediator* development.

During the egg stage, 12 RR‐1, RR‐2, and all CPAP3 and CPAP1 CP genes had relatively high FPKM. During larval development, the expression pattern of CP genes had major changes. The majority of transcripts that were highly expressed during the embryo stage were down‐regulated during larval development. The number of CP genes with detectable expression levels decreased to 23 in 1st instar larvae. A total of 43% of these belonged to the RR‐1 subfamily. Compared to egg transcript abundance, the only protein with an up‐regulated expression pattern in the 1st instar was *MmCPAP1‐J*. All others maintained the egg expression level (*MmCPR1*, *2*, *3* and *4*) or had decreased expression (the majority of the CP genes). During the 2nd instar, 26 CPs had relatively high transcript abundance, and 10 of these were in the RR‐1 family. Four CP transcripts (*MmCPR1*, *2*, *3* and *4*) also had high expression levels during the 2nd instar. *MmCPR5*, *MmCPAP1‐N* and *MmCPLCP‐1* had increased transcript abundance compared to eggs and 1st instars. Expression of other genes was down‐regulated compared to the 1st instar. The 3rd instar had detectable transcript levels of 26 CPs. *MmCPR6* and *MmCPR14* genes had their expression peaks during this stage. During late larval development, only 12 CP protein genes had relatively high expression. Seven of these belonged to RR‐1 (*MmCPR1*, *2*, *3*, *5*, *6*, *13* and *14*), CPAP3 (*MmCPAP3‐C*), CPAP1 (*MmCPAP1‐N, ‐H*), TWDL (*MmTWDL‐1*), and CPLCP (*MmCPLCP‐7*). The pupa had a high number of CP genes with relatively high transcript levels, and 74% of all annotated CP genes were up‐regulated during this stage. Apidermin and CPLCP families, *MmCPR12* and *MmCPR37*, appear to be stage‐specific with high expression limited to the pupal stage. During the adult stage, only 24 CP genes were transcriptionally active. A limited number of proteins had high expression levels during the larval stages. Most of these belonged to the RR‐1 subfamily.

**Figure 6:**
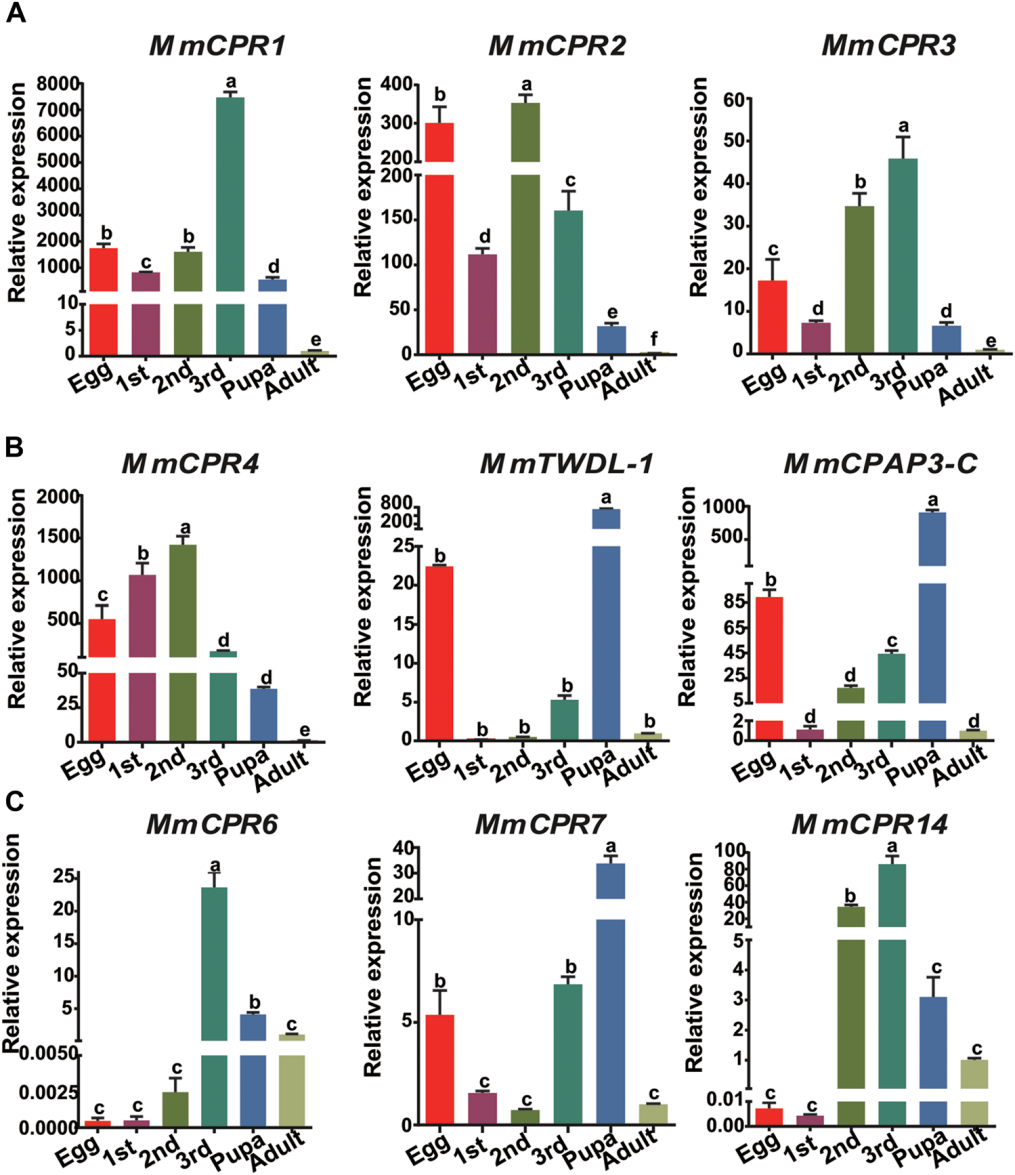
Relative expression level of *Microplitis mediator* cuticular protein (CP) genes during developmental stages. (A) CP genes *MmCPR1*, *MmCPR2*, *MmCPR3*, *MmCPR4*, *MmTWDL* and *MmCPAP3‐C* are characterized by a high expression level during egg and larval developmental stages. (B) CP genes *MmCPR7*, *MmCPR6* and *MmCPR14* are characterized by a high expression level during late larval and early pupal developmental stages. The expression of CPs was analyzed by quantitative polymerase chain reaction (qPCR), cDNA templates were prepare from total RNA isolated from egg, 1st, 2nd, 3rd instar larvae, pupae, and adults. β‐actin gene was used as an internal quantitative control. *M. mediator* adult samples were accepted as relative samples. Different letters above deviation bars indicate statistical difference between different stage values.

Only a few CP genes were activated during the late development of parasitoid larvae inside the host, and these were studied in more detail. To confirm the developmental expression pattern, nine of the CP genes with high transcript abundance in 1st, 2nd, and 3rd instar larvae were chosen for qPCR. Based on their expression patterns, the CPs could be divided into two groups. The first group was activated during the egg stage and maintained expression during larval development (Fig. 6A). This group includes *MmCPR1*, *MmCPR2*, *MmCPR3*, *MmCPR4*, *MmCPAP3‐C*, and *MmTWDL‐1* CP genes. The second group of proteins had an expression peak during the late larval or early pupal stages (Fig. 6B). It includes *MmCPR7*, *MmCPR6*, and *MmCPR14*.

### Functional analysis of M. mediator larval CPs

**Figure 7:**
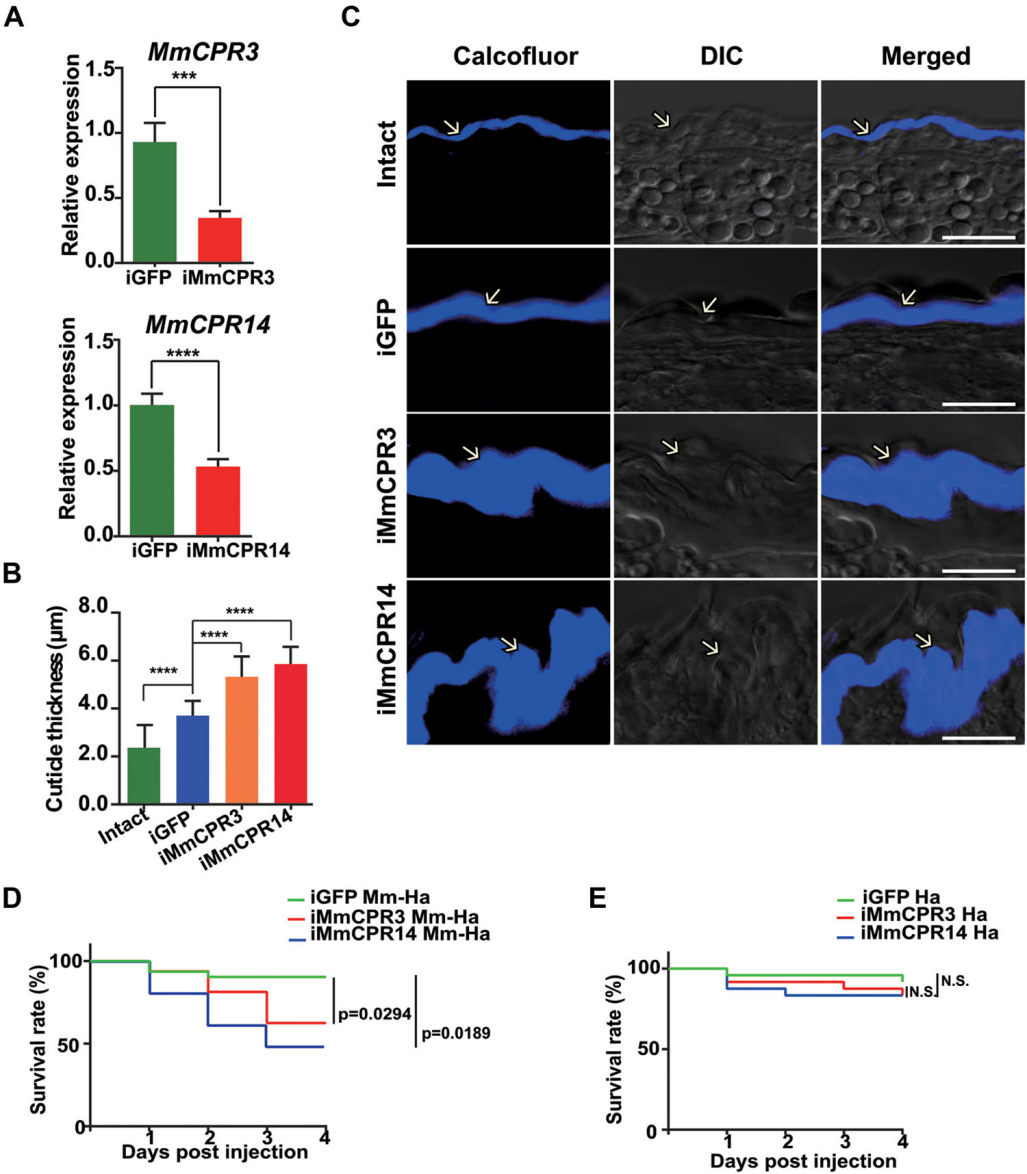
Functional analysis *Microplitis mediator* larval cuticular proteins (CPs) MmCPR3 and MmCPR14. (A) Relative quantification of *MmCPR3* and *MmCPR14* expression level after injection of corresponding double‐stranded RNA (dsRNA). Double‐stranded green fluorescent protein (dsGFP)‐injected insects were treated as control samples. Columns represent the mean value of three replicates. Statistical significance values (one‐way analysis of variance): injected GFP (iGFP) vs. iMmCPR3 (*P* = 0.0007), and iGFP vs. iMmCPR14 (*P* < 0.0001). (B) Cuticle thickness in *MmCPR3* or *MmCPR14*‐depleted insects, non‐treated and dsGFP‐treated *M. mediator*. All morphometric measurements were performed using ImageJ. Columns represent mean cuticle thickness in µm with standard deviation bars. Statistical significance values (Mann–Whitney test): intact vs. iGFP (*P* = 0.0001), iGFP vs. iMmCPR3 (*P* < 0.0001), and iGFP vs. iMmCPR14 (*P* < 0.001). (C) Chitin‐specific staining of 3rd instar *M. mediator* larvae injected with dsMmCPR3, dsMmCPR14 or dsGFP. Non‐treated controls were provided. Samples for cryosectioning were obtained on the third day after dsRNA injection and 7 µm sections were used for further staining. Cuticle chitin was detected with fluorescence dye Calcofluor White M2R. Scale bars: 10 µm. Microphotographs were chosen as representations of full results. (D) Kaplan–Meier survival curves of parasitized *Helicoverpa armigera* (Ha‐Mm) treated with dsMmCPR3 or dsMmCPR14 and control parasitized *H. armigera* treated with dsGFP. Significant differences were labeled. (E) Kaplan–Meier survival curves of non‐parasitized *H. armigera* (Ha) treated with dsMmCPR3 or dsMmCPR14 accordingly and control *H. armigera* treated with dsGFP. N.S. indicates no significant differences between the control and experimental groups.

Two CPs MmCPR3 and MmCPR14 were chosen for further functional analysis. The expression of *MmCPR3* gene was high during all larval stages, while *MmCPR14* gene was activated during 3rd instar larvae in contrast to other CPs. To investigate their functions in development, we knocked down the expression of these genes using dsRNA‐mediated silencing. Since *M. mediator* is an obligatory endoparasitoid wasp with larval development inside of the host body, we injected dsRNA into the *H. armigera*‒*M. mediator* parasitoid system. Injection of dsMmCPR3 or dsMmCPR14 significantly decreased the corresponding transcripts. This was confirmed using qPCR (Fig. 7A). To check whether MmCPR14 and MmCPR3 are essential for parasitoid larval development, we performed survival curves analysis. Since it is impossible to calculate *M. mediator* survival without dissection from the host body, we calculated the survival of the entire parasitoid system of *M. mediator*‒*H. armigera* that ended with parasitoid wasp emergence from the host body at the fourth day after injection. Injection of dsMmCPR3 or 14 caused high mortality of the entire *H. armigera*‒*M. mediator* parasitoid system compared to the control (Mm iMmCPR3 vs. Mm iGFP: *P *< 0.0294, Mm iMmCPR14 vs. Mm iGFP: *P* < 0.0189) (Fig. 7D).

To exclude the interference of dsRNA on *H. armigera* development, we injected dsMmCPR3 or 14 into non‐parasitized *H. armigera*. There was no significant effect on *H. armigera* larval survival compared to dsGFP‐injected non‐parasitized larvae (Fig. 7E). The synthesized dsRNA used did not interfere with host CPs but appeared to specifically target *M. mediator MmCPR3* or *14* CP genes. These data suggested that the injection of dsMmCPR3 or 14 has an exclusive effect on parasitoid wasp development. However, developmental retardation and/or parasitoid larvae death did affect the survival of the entire parasitoid system. Dead hosts had a reduced body size and darker coloration, possibly due to water loss (Fig. S2 A).

On the third day after parasitization, *M. mediator* from surviving hosts showed no detectable phenotypic abnormalities compared to the control group. Body structure, size, and coloration were not affected by *MmCPR3* or *14* deficiency. *M. mediator* dissected from dead hosts showed no movement. Based on body size and coloration, it appeared that development cessation of 2nd instar *M. mediator* led to the death of the host (Fig. S2 B).

To study the role of MmCPR3 and MmCPR14 in the organization of cuticle structure, we prepared cryosection slices for staining. The histological samples were taken on the third day after injection. For confocal observation, we used fluorescent dye Calcofluor, which has known chitin‐biding abilities. In *MmCPR3* or *14*‐depleted *M. mediator*, the thickness of cuticle visibly increased compared to the control tissues of GFP‐injected and non‐treated *M. mediator* (Fig. 7C). The chitin thickness of the control and the treated insects was estimated by measuring all cuticle layers using ImageJ software. The larval CPs knock‐down of *M. mediator* caused a significant increase of chitin layer thickness in both treatment groups. Chitin thickness increased from 2.495 ± 0.80 *µ*m (intact) and 3.68 ± 0.67 *µ*m (dsGFP) in the control groups to 5.31 ± 0.85 *µ*m and 5.85 ± 0.71 *µ*m in *MmCPR3*‐ or *MmCPR14*‐deficient insects, respectively (*P* < 0.0001) (Fig. 7B).

## Discussion

The development of *M. mediator* is dependent on host type and stage of development, temperature and photoperiod (Khan, 1999; Foerster, 2003; Li *et al*., 2008). The complete life cycle of *M. mediator* requires 24 days (Arthur & Mason, 1986). The parasitoid female penetrates the host integument with the ovipositor and injects egg and venom. After hatching from the egg, the larvae become hemolymph feeders. Late 3rd instar larvae emerge from the host body and pupate. After several days, the adult wasp emerges (Arthur & Mason, 1986; Khan & Özer, 1988). Transcriptomic libraries that cover all developmental stages of *M. mediator* described above (Fig. S1) were used for CPs annotation.

Transcriptome‐based analysis revealed 70 putative CPs. The number of CPs is similar to that found in other Hymenoptera species, such as *A. mellifera* (45 CPs) (Consortium, 2006; Micas *et al*., 2016) and *N. vitripennis* (76 CPs) (Werren *et al*., 2010; Willis, 2010). However, the number of CPs in *M. mediator* is lower than some non‐Hymenoptera species (Dittmer *et al*., 2015; Yang *et al*., 2017). The low number of annotated CPs in Hymenoptera species could be explained by their highly developed social organization (*A. mellifera*) or parasitic lifestyle during early developmental stages (*N. vitripennis*). During the larval period of *M. mediator*, larvae may not need a strong, protective exoskeleton. The host cuticle could help protect parasitoid larvae from harmful environmental factors.

CPR is the most numerous family in *M. mediator*. It possesses the RR chitin‐binding motif, which distinguishes it from other CPs. A total of 16 RR‐1 CPs were identified in *M. mediator*. The biggest subfamily was RR‐2, and it consists of 23 putative proteins. A similar number of RR‐2 was found in *M. sexta*, *B. mori*, and *Anopheles gambiae* and consisted of 60%‒65% of the annotated CPR number (Cornman *et al*., 2008; Futahashi *et al*., 2008; Dittmer *et al*., 2015). For the *M. mediator* transcriptome, this ratio is 58% of the discovered CPR proteins. Physical characteristics of the cuticle could be dominant factors of RR‐1 and RR‐2 protein localizations (Vannini & Willis, 2017). RR‐1 is usually associated with flexible and less sclerotized layers of cuticle, such as hind wings and intersegmental membrane (Andersen, 2000). However, RR‐2 is associated with rigid regions of cuticle, such as head capsules (Dittmer *et al*., 2012; Zhou *et al*., 2016). The high levels of histidine and lysine amino acids provide possible targets for the sclerotization of RR‐2‐rich cuticle layers (Shahin *et al*., 2018).

Phylogenetic analysis of CPR proteins revealed several proteins that seem to be species‐specific and clustered separately from *A. mellifera* and *N. vitripennis* CPR. These include MmCPR1, 3 and 14 (RR‐1); and MmCPR20, 22, 23, and 24 (RR‐2). This group of CPR proteins could be specific for *M. mediator*. CPR members with proven physiological functions from other species did not provide help in finding clear orthologs. Further investigation will be required to clarify their role in cuticle formation and development.

CPAP is the second largest protein group in *M. mediator*. The conservative structure of CPAP ChtBD can be described by the six cysteines motif with variable amino acids inserted between them (Figs. 3B and 4B). This protein group is required for normal insect molting, development, locomotion, and preservation of the cuticle integrity (Jasrapuria *et al*., 2012; Petkau *et al*., 2012; Pesch *et al*., 2015). Expression analysis of *M. mediator* CPAP1 and CPAP3 families revealed specificity for earlier developmental stages from egg to pupa. Only MmCPAP1‐H maintained its earlier expression level in adults. The number of CPAP representatives in the *M. mediator* transcriptome (12 CPAP1 and 5 CPAP3) is similar to other insects (*T. castaneum* 10 CPAP1 and 7 CPAP3; *M. sexta* 15 CPAP1 and 10 CPAP3 proteins) (Jasrapuria *et al*., 2010; Tetreau *et al*., 2015). The similarity in protein number indicates relatively conservative functions of this family and less sequence variability.

Based on phylogenetic analysis, we divided CPAP1 and CPAP3 CPs into different groups. The 12 CPAP1 proteins were not evenly divided. Among 16 groups, only nine (N, K, H, B1, B2, M, F, G, and I) were represented in *M. mediator* (Fig. 3A). Other groups were not identified in this study. These results differ from *M. sexta* CPAP1 proteins that have been divided into almost every group of CPAP1 phylogenetic clades, except for E (Tetreau *et al*., 2015). Functional analysis of *T. castaneum* CPAP1 proteins (Jasrapuria *et al*., 2012) revealed that only three representatives of CPAP1 were indispensable for normal insect development, although only the H group was annotated in this study. Knock‐down of the CPAP1 gene from the H group in *T. castaneum* decreased elytra chitin thickness. MmCPAP1‐H may play a similar role in designating cuticle thickness.

The CPAP3 family of *M. mediator* CPs is distributed among five groups: C, D1, D2, A1, and B (Fig. 4A). Proteins from A2 and E were not found among annotated CPs in this study. Injection of the dsRNA of TcCPAP3‐A2 or TcCPAP3‐E had no visible effect on *T. castaneum* molting and cuticle thickness (Jasrapuria *et al*., 2012). This may be why the analogs of these proteins were not found in this study. Knock‐down of the other CPAP3 caused severe molting and development defects. This group is associated with insect adaptation to severe environmental conditions (Chen *et al*., 2018). The protection afforded by the host cuticle during the developmental stages of *M. mediator* might decrease the necessity of these CPs.

The remaining families of CP have a much lower number of proteins. However, some of these are required for normal insect development. Analysis of transcriptomic databases allowed the annotation of 13 CPs that were divided into four families, TWDL, CPLCP, CPF, and Apidermin. The TWDL family of CPs is important for maintaining normal body shape during larval development (Guan *et al*., 2006). Two proteins from the TWDL family were annotated in this study. This number is lower compared to *D. melanogaster* but similar to other Hymenoptera species (*A. mellifera* and *N. vitripennis*). CPLCP is a CPs family enriched in proline, valine, lysine, and tyrosine residues (Cornman & Willis, 2009). In the *M. mediator* transcriptome, we annotated seven proteins from the CPLCP family, and this number is higher than in other Hymenoptera species.

Another annotated CP family is CPF, the functions and localization of which remain unclear (Togawa *et al*., 2007). Since their chitin‐binding capability is unproven, they could be considered as epicuticle components that lack chitin fibrils (Papandreou *et al*., 2010). The Apidermin family is common in Hymenoptera species and was annotated in *A. mellifera* and *Nasonia* (Kucharski *et al*., 2007; Werren *et al*., 2010; Micas *et al*., 2016). Proteins of this family are formed from highly hydrophobic polypeptides with a high content of nonpolar amino acids such as alanine (the most abundant), glycine, leucine, proline, and valine. We annotated three representatives of this CP family in the *M. mediator* transcriptome, and it is similar to other Hymenoptera.

Expression analysis of CP genes revealed two expression peaks during egg and pupa developmental stages. During these periods, a large number of CPs were expressed. Embryonic and pupal time points appear to be crucial for normal cuticle formation and development of *M. mediator*. Their expression was associated with body morphology plan and shape, thickness of cuticle layers, and symmetry. Expression of these CPs is indispensable for further development (Kotze & Reynolds, 1990; Guan *et al*., 2006; Qiao *et al*., 2014).

CP expression levels in *M. mediator* larvae are greatly down‐regulated compared to the egg stage. A similar fluctuation in expression was observed during ontogenesis of *Pieris rapae* (Qi *et al*., 2016) and *Cnaphalocrocis medinalis* (Li *et al*., 2012). The expression pattern was reversed during pupal development. Numerous CP transcripts were up‐regulated, compared to final instar larvae. This activation pattern is similar to *Athetis lepigone* (Li *et al*., 2013) and *Spodoptera litura* (Gu *et al*., 2013) CPs. The number of transcriptionally activated CP genes decreased from the pupal to the adult stage, and this is consistent with the expression profile of *B. mori* wing discs (Shahin *et al*., 2018). The embryonic and pupal time points appear to be crucial for normal cuticle development. Between these time points, expression of a limited number of CPs assures normal growth and larval molting of *M. mediator*. This gene cohort could be essential for normal cuticle formation and molting processes inside of the host body. The majority of transcriptionally active genes belong to the RR‐1 subgroup, and they are associated with soft cuticle (Zhou *et al*., 2016). Formation of soft and flexible cuticle during larval development stages of *M. mediator* could facilitate successful emergence from the host body and pupation.

Hierarchical clustering analysis indicated that the majority of *M. mediator* CPs possess stage‐specific expression peaks. A few CPs are common for both the egg and pupal stages. However, the possibility of CP stage specificity remains controversial. Studies on *A. gambiae* revealed 25 CPs with possible specificity to a single developmental stage. Approximately 10% of all CPs of *A. gambiae* may have specific functions for particular developmental stages (Togawa *et al*., 2008). High stage specificity of *M. mediator* CP expression peaks could be due to the parasitoid life cycle where the developing larvae require a special cuticle composition. Another explanation is that the undetectable levels of CPs between larval molts are due to different hormonal activating mechanisms of pre‐molt and pharate larval CPs.

Because of the large number of CP genes identified in *M. mediator*, the specific role of each protein will require further study. Currently, only one study has summarized the deficiency phenotypes of all CPs annotated in *Nilaparvata lugens* (Pan *et al*., 2018). Others studies have focused on the most abundant CPs during specific developmental periods (e.g., adult molting) or a specified body part such as the wing disc (Noh *et al*., 2014; Qiao *et al*., 2014; Chandran *et al*., 2016).

The study of *M. mediator* larval development is challenging since it entirely occurs inside the host. Because *M. mediator* larvae are hemolymph feeders (Dahlman & Vinson, 1980), we postulated that injection of dsRNA into the host cavity would be an effective technique for functional studies of parasitoid wasp CPs. Ingested dsRNA can lead to a systemic RNAi reaction in non‐gut tissues (Yu *et al*., 2013). For example, silencing of chitin synthase A, specifically expressed in cuticular and tracheal epidermis, was demonstrated in *S. exigua* (Tian *et al*., 2009). Gene suppression in the eastern subterranean termite, *Reticulitermes flavipes*, occurred in gut tissues (cellulase gene Cell‐1 and hexamerin gene Hex‐2) and also in the salivary glands (Cell‐1) and fat body (Hex‐2) (Zhou *et al*., 2008). In contrast, some insect species showed no, or only a weak, response to dsRNA feeding. These include several *Drosophila* species, *Rhodnius prolixus*, and *S. litura* (Rajagopal *et al*., 2002; Araujo *et al*., 2006; Whyard *et al*., 2009). Gene suppression may depend on gut conditions that vary among insect species (Yu *et al*., 2013). In Hymenoptera species, several studies have used ingestion of dsRNA for silencing of endogenous genes as well as for virus‐specific genes in *A. mellifera* and *B. terrestris* (Hunter *et al*., 2010; Piot *et al*., 2015; Li *et al*., 2016). Several mechanisms of dsRNA cell uptake have been discovered (Winston *et al*., 2002; Saleh *et al*., 2006), but dsRNA translocation from the digestive tract remains poorly understood. Another possible mechanism to deliver dsRNA from the host hemolymph to *M. mediator* larvae is soaking through the cuticle. However, this method is mostly used for cell culture transfection (Clemens *et al*., 2000). Some reports showed successful dsRNA uptake by the *Drosophila* embryo and by the newly hatched larvae of *Ostrinia furnacalis* through its cuticle (Yuen *et al*., 2008; Wang *et al*., 2011). However, the mechanism by which dsRNA entered the body of *M. mediator* remains undetermined.

Knock‐down of *M. mediator* larval CPs caused high mortality of the parasitized *H. armigera*, and this could not be explained by dsRNA co‐interference with the host larvae CPs (Fig. 7D and E). Possible causes include the disruption of hormone titer due to parasitic factors or inflammation caused by decaying parasitic larvae. The injection of larva CPs dsRNA did not lead to visible abnormalities in external morphology or molting of surviving *M. mediator* emerging from the host body (Fig. S2 B), and this is consistent with previous reports (Mun *et al*., 2015; Chandran *et al*., 2016). However, other studies reported a change in body shape, coloration, or leg structure after the silencing of particular CPs (Qiao *et al*., 2014; Mun *et al*., 2015; Xiong *et al*., 2017). To study the function of selected larval CPs, we performed a microstructural analysis.

The most common phenotype in insects deficient in one or more CPR proteins is disorganization of chitin fibrils and lamina and changes in the physical properties of the cuticle (Noh *et al*., 2014; Chandran *et al*., 2016). Depletion of two RR‐1 larval CPs in *M. mediator* led to a similar phenotype. This result is consistent with previous studies. Insects lacking normal levels of *MmCPR3* or *MmCPR14* transcripts had an increased chitin layer, which may have resulted from abnormalities in chitin fibrils organization. Due to the limited number of highly expressed proteins during larval development, every distinct CP may be important in the cuticle organization of late‐instar *M. mediator* larvae.

Parasitoid wasp larvae develop under unique conditions inside their host. They undergo two larval molts, and the 3rd instar larvae emerge to pupate (Arthur & Mason, 1986). During parasitization, host developmental processes are blocked by several mechanisms. As developmental stages and cuticle formation dynamics affect CP expression, study of *M. mediator* CP could clarify the survival and developmental strategies of parasitoid larvae. The small group of CPs that are activated during late larval development of *M. mediator* would be interesting targets for further investigation.

In conclusion, 70 putative CPs were identified in the *M. mediator* transcriptome that includes the developmental stages from egg to adult. All sequences were manually annotated and checked for ChtBD or specific consensus possession. This allowed division of the sequences into seven distinct CP families. Hierarchical clustering analysis of annotated CPs revealed a small number of CP genes that were transcriptionally active during the late larval stages, which suggested their critical roles in development. Functional analysis of two larval CPs, MmCPR3 and 14, showed their important functions in normal cuticle formation and larval survival.

## Disclosure

The authors have no conflicts of interest.

## Supporting information


**Fig. S1**. Images of *Microplitis mediator* showing the morphology of main developmental stages that were used to prepare RNA‐libraries. (A) Egg, (B) 1st instar, (C) 2nd instar, (D) 3rd instar, (E) pupa, and (F) adult.

**Fig. S2**. Effect of dsMmCPR3, 14, or double‐stranded green fluorescent protein (dsGPF) injection on host‐parasitoid phenotype 3 days after injection. (A) Photograph of parasitized hosts (Ha‐Mm) exposed to treatment with dsMmCPR3, 14, or dsGPF. (B) Photograph of *Microplitis mediator* (Mm), that were treated with dsMmCPR3, 14, or dsGPF.

**Table S1**. *Microplitis mediator* cuticular protein (CP) sequence characteristics and fragments of kilobase per transcript per million fragment mapped (FPKM) values during major developmental stages.

**Table S2**. List of primers used in quantitative polymerase chain reaction and RNA interference experiments.

**Table S3**. Amino acid sequences of *Microplitis mediator* cuticular proteins (CPs).

**Table S4**. Accession numbers of cuticular protein (CP) genes, used in phylogenetic analysis and Rebers and Riddiford (RR)‐3 proteins annotation.
