# Supplementary material for: Identification and temporal expression profiles of cuticular proteins in the endoparasitoid wasp, Microplitis mediator
Source: Insect Sci. 2019 Aug 6;27(5):998–1018. doi: 10.1111/1744-7917.12711 (PMC7497268; doi:10.1111/1744-7917.12711)
Supplement: Supplementary file 3 — Table S1. Microplitis mediator cuticular protein (CP) sequence characteristics and fragments of kilobase per transcript per million fragment mapped (FPKM) values during major developmental stages. [file INS-27-998-s003.pdf]

Table S1. ***M. mediator*** **CP sequences characteristics and FPKM values during major developmental stages.**

Gene	Signal peptide	Full length	Length	FPKM egg	FPKM 1st instar	FPKM 2nd instar	FPKM 3rd instar	FPKM pupa	FPKM adult	Expression cluster
MmCPR1	Y	Y	131.00	1728.79	537.67	351.37	487.36	7.20	1.19	III
MmCPR2	Y	Y	138.00	756.65	310.17	220.88	74.55	2.78	0.70	III
MmCPR3	Y	Y	105.00	939.92	500.70	207.95	321.64	0.99	6.77	III
MmCPR4	Y	Y	133.00	444.53	369.63	138.73	10.68	18.55	4.80	III
MmCPR5	Y	Y	193.00	78.73	0.64	130.84	117.51	529.65	34.11	I
MmCPR6	Y	Y	166.00	0.83	0.23	0.87	243.15	13.84	6.89	IV
MmCPR7	Y	Y	168.00	130.01	12.74	10.43	7.21	238.81	13.08	I
MmCPR8	Y	Y	147.00	369.64	92.29	17.52	1.14	285.70	378.43	I
MmCPR9	Y	Y	133.00	28.23	4.66	0.36	0.00	94.06	482.12	I
MmCPR10	Y	Y	159.00	747.48	147.82	2.58	0.50	8.20	0.90	II
MmCPR11	Y	Y	190.00	1175.39	5.65	21.09	11.29	1144.43	1.15	I
MmCPR12	Y	Y	146.00	5.49	0.39	0.17	6.89	194.95	19.55	I
MmCPR13	Y	Y	104.00	291.04	100.62	6.88	42.59	257.77	82.54	I
MmCPR14	Y	Y	176.00	183.48	20.15	8.85	254.66	0.82	8.58	IV
MmCPR15	Y	Y	310.00	2.38	0.31	0.00	0.06	47.41	4.41	I
MmCPR16	Y	Y	207.00	0.00	0.18	0.00	0.00	24.47	78.76	I
MmCPR17	Y	Y	348.00	8.86	0.49	0.73	0.34	89.18	0.18	I
MmCPR18	Y	Y	139.00	1.98	0.13	0.11	0.17	1.01	2.03	II
MmCPR19	Y	Y	131.00	72.08	0.29	0.00	0.38	0.47	0.39	II
MmCPR20	Y	Y	293.00	1.44	0.06	4.85	17.94	1.83	0.46	IV
MmCPR21	Y	Y	182.00	36.81	1.46	1.46	0.54	121.05	0.09	I
MmCPR22	Y	Y	252.00	0.41	0.15	2.20	3.59	0.16	0.13	IV
MmCPR23	Y	Y	228.00	0.57	0.08	2.22	2.16	0.18	0.30	IV
MmCPR24	Y	Y	151.00	38.77	2.65	0.08	0.07	0.00	0.08	II
MmCPR25	Y	Y	174.00	1778.38	215.35	1.53	2.43	15.46	3.51	II

MmCPR26	Y	Y	238.00	17.38	0.32	0.86	0.83	130.38	0.11	I
MmCPR27	Y	Y	231.00	8.86	0.49	0.73	0.34	89.18	0.18	I
MmCPR28	Y	N-terminal	138.00	1838.08	94.28	10.08	1.07	0.90	0.86	II
MmCPR29	Y	N-terminal	147.00	1308.10	105.62	6.29	1.40	1.54	0.43	II
MmCPR30	Y	N-terminal	178.00	45.81	0.11	0.81	1.43	235.38	4.55	I
MmCPR31	Y	Y	198.00	38.56	0.10	12.43	2.19	3.55	0.21	II
MmCPR32	Y	Y	230.00	47.76	0.83	0.42	0.17	48.92	1.83	I
MmCPR33	Y	Y	140.00	0.49	0.54	0.00	0.00	1.33	0.97	I
MmCPR34	Y	Y	130.00	0.00	0.00	0.00	0.00	15.47	0.03	I
MmCPR35	Y	Y	202.00	0.21	0.00	0.00	0.00	7.52	4.84	I
MmCPR36	Y	Y	138.00	5.31	2.90	1.40	1.72	4.13	18.21	I
MmCPR37	N	C-terminal	203.00	1.27	0.00	0.48	0.00	31.83	0.62	I
MmCPR38	N	C-terminal	226.00	1.98	0.13	0.11	0.17	1.01	2.03	I
MmCPR39	Y	Y	198.00	39.91	18.19	4.88	21.67	94.93	30.75	I
MmCPR40	Y	Y	287.00	5.18	0.93	0.00	0.27	1.65	0.79	II
MmCPAP1-N	Y	Y	214.00	1.37	0.18	64.89	23.12	1.35	0.08	IV
MmCPAP1-H	Y	Y	192.00	140.00	14.75	30.93	26.61	145.79	11.50	I
MmCPAP1-K1	Y	Y	607.00	22.77	0.62	4.04	0.45	10.02	3.49	II
MmCPAP1-B2	Y	Y	692.00	26.05	2.59	3.16	3.44	24.89	0.61	I
MmCPAP1-I	Y	N-terminal	187.00	167.08	14.56	12.84	5.76	181.75	0.36	I
MmCPAP1-1	Y	N-terminal	267.00	6.16	25.36	0.36	2.09	9.28	2.07	I
MmCPAP1-M1	Y	N-terminal	247.00	10.68	0.92	1.07	0.67	11.29	1.26	I
MmCPAP1-M2	Y	N-terminal	251.00	7.61	1.02	1.72	0.86	12.66	0.85	I
MmCPAP1-K2	Y	Y	562.00	22.46	0.66	3.72	0.54	9.31	3.47	II
MmCPAP1-B1	Y	Y	220.00	13.81	11.31	2.30	2.50	8.17	0.67	III
MmCPAP1-F	Y	Y	124.00	53.69	0.77	1.07	0.00	144.51	5.90	I
MmCPAP1-G	Y	Y	331.00	340.49	10.37	8.51	2.97	1240.66	0.77	I
MmCPAP3-C	Y	Y	266.00	1016.48	24.30	25.51	19.40	252.59	1.84	II
MmCPAP3-D2	Y	Y	257.00	360.26	26.52	11.12	3.63	263.92	4.55	I
MmCPAP3-B	Y	Y	292.00	105.28	0.52	3.14	8.82	34.78	0.35	II

MmCPAP3-D1	Y	N-terminal	229.00	270.56	16.35	1.36	0.51	50.27	2.01	II
MmCPAP3-A1	Y	Y	232.00	889.19	23.91	43.41	29.59	695.35	2.52	I
MmTWDL-1	Y	Y	184.00	401.94	1.71	30.64	27.40	517.54	0.80	I
MmTWDL-2	Y	Y	286	59.19	5.87	2.65	1.22	2.55	0.03	II
MmCPLCP-1	Y	Y	378.00	4.48	2.16	16.34	8.16	5039.96	0.47	I
MmCPLCP-2	Y	Y	140.00	11.21	0.14	4.23	0.28	112.86	0.06	I
MmCPLCP-3	Y	N-terminal	261.00	1.84	0.72	2.39	1.58	1384.79	0.29	I
MmCPLCP-4	Y	Y	221.00	10.82	0.00	2.13	2.23	95.99	0.08	I
MmCPLCP-5	Y	N-terminal	529.00	0.03	0.00	0.09	0.07	36.99	0.09	I
MmCPLCP-6	Y	N-terminal	137.00	1.94	0.21	0.18	2.22	77.41	0.21	I
MmCPLCP-7	Y	N-terminal	385.00	30.54	3.07	13.32	46.62	3010.03	2.28	I
MmCPF-1	Y	Y	203.00	3660.91	2.43	40.24	3.98	9.47	0.33	II
MmApd-1	Y	Y	283.00	18.41	3.15	7.75	4.24	8474.84	21.85	I
MmApd-2	Y	Y	167.00	0.00	0.00	0.00	0.00	0.87	15.51	I
MmApd-3	N	C-terminal	265.00	0.45	0.36	0.00	0.07	18.55	3.18	I
