# Supplementary material for: Identification and temporal expression profiles of cuticular proteins in the endoparasitoid wasp, Microplitis mediator
Source: Insect Sci. 2019 Aug 6;27(5):998–1018. doi: 10.1111/1744-7917.12711 (PMC7497268; doi:10.1111/1744-7917.12711)
Supplement: Supplementary file 4 — Table S2. List of primers used in quantitative polymerase chain reaction and RNA interference experiments. [file INS-27-998-s004.pdf]

Table S2. List of primers that were used in qPCR and RNAi experiment.

Gene	Experiment	Primer sequence (5'-3')	Forward/ Reverse
MmCPR1	qRT-PCR	GCACAGCAATACAACCAGCA	F
		CGGGTTGTCCTTGTTCGTCA	R
MmCPR2	qRT-PCR	GTGCGTCAGAAAACAGCAAA	F
		TTCTGGGATTGGAGGAGATG	R
MmCPR3	qRT-PCR	TGAAAATGATAATCGCACTCGT	F
		CTCCGATGTTGTTGTTCTCTTG	R
MmCPR4	qRT-PCR	TAGCAGCACCACAGTACCAG	F
		GAACCCTGCTCTCGTGCTAG	R
MmTWDL-1	qRT-PCR	TCATGTACCTCCCCCAGAAG	F
		GGGGCTGTACGGGAATTACT	R
MmCPAP3-C	qRT-PCR	CCGAAGATGTTCCTGGATGT	F
		CTCGATGACACGTGATTTGG	R
MmCPR6	qRT-PCR	TCCACAATGTCTGGGAATCA	F
		GCCACATAAGTCAGCGTGAA	R
MmCPR7	qRT-PCR	CTGACGAAGCCCAACTGATT	F
		CCCTTTGAATTTCTGGTGGA	R
MmCPR14	qRT-PCR	ATTCCTTGTCGTTGCCTTTG	F
		GGTGATTGATCCTGGGATTG	R
β-actin	qRT-PCR	GGCCCCATCAACCATCAAGA	F
		GGACCGGATTCGTCGTACTC	R
MmCPR3	dsRNA	TAATACGACTCACTATAGGGGATGGA TACCAGTACAGCTACGAA	F (with adaptor)
		TAATACGACTCACTATAGGGTCAAGC AGGGATGTGTGC	R (with adaptor)
MmCPR3	dsRNA	GATGGATACCAGTACAGCTACGAA	F
		TCAAGCAGGGATGTGTGC	R
MmCPR14	dsRNA	TAATACGACTCACTATAGGGACACCG ATAACGGAATCCAC	F (with adaptor)
		TAATACGACTCACTATAGGGCCTTGA GGTGTTGGAGCAGT	R (with adaptor)
MmCPR14	dsRNA	ACACCGATAACGGAATCCAC	F
		CCTTGAGGTGTTGGAGCAGT	R
