# Supplementary material for: Identification and temporal expression profiles of cuticular proteins in the endoparasitoid wasp, Microplitis mediator
Source: Insect Sci. 2019 Aug 6;27(5):998–1018. doi: 10.1111/1744-7917.12711 (PMC7497268; doi:10.1111/1744-7917.12711)
Supplement: Supplementary file 5 — Table S3. Amino acid sequences of Microplitis mediator cuticular proteins (CPs). [file INS-27-998-s005.pdf]

Table S3. Amino acid sequences of *M. mediator* CPs

Name	Accession number	Amino acid sequence
MmCPR1	MK632949	MKFLIISLAVIAAVSAQQYNQQNQQPIAIVRQSQDSSPDGSYQYSYETEN<br>GISVDEQGQPVAQGPDGPAVVAQGGYQYVGDDGVTYQVRYTADETG<br>FHPEGAHLPVSPPIPEAIARALEYIRTHPQPQEQ-
MmCPR2	MK632950	MYKLAVILGLVSMVLAAPVDQQSTSEIPIVRESHDGPNPDGSYSWSYEA<br>GNGIQAQEEGSLSASENSKDGALVVRGSYSYTGEDGVQYQIVYVADEN<br>GFQPQGAHLPTSPPIPEEILKSLEYNAAHPEENNDDEPKQ-
MmCPR3	MK632951	MKMIIALVALVAVAAAAPVEDVVVVKSQENNNIGVDGYQYSYELSNG<br>QSVQEVGELRNVPGEESPVVVVKGSFSYTDPATGTQYTVTYVADENGF<br>QPSGAHIPA-
MmCPR4	MK632952	MFSKALIVLAIVAAALAAPQYQSESSTPIPIISESRNGPNPDGSYSWSYET<br>ANGILAREQGSLVQSDNKEALSVSGGYKYIGEDGNPIEITYTADENGYQ<br>PQGAHLPVAPPIPEAILKALEWIAAHPEEDRL-
MmCPR5	MK632953	MKFLIISFAIISCAFAQYGGGFRPFPKPAITPKPYPIPQPQPQFPSQFQPQQ<br>PQYQPQNQFRPSGPIIPIRSQSQDSSPDGSYQFSYETDNGISVSETGSPKG<br>VATPDGQAEVVQGRYSYVAPDGTPITVEYYADETGFHATGAHIPTPPPI<br>PEAIQRAIASLPPSNEGQYQEEQYNNRQYNQNPGGPFRRF-
MmCPR6	MK632954	MSKIILFALSVTIAQSAKLTNAPEYRQVQNPTHQDHVPVHHQDHTMINK<br>PVGILNQESEINPDGSFHNVWESENGIKVQEEGTVRELAKNVVAHTVTG<br>RIAYTDNEDNVFTLTYVADENGFRPEGSHLPTPPPIPLAIARALEYIAAHP<br>SETNSAHKDEPVVEAN-
MmCPR7	MK632955	MRVILVAATILSVTSGQEYFREPEKIISDSRQVGDKLGQYSVAYQTDGGI<br>LQKEVGSRKYAGTADEAQLIQGSVQYNAPDGTPVSFSWSADEFGTQIH<br>GTHIPTPPPIPPEIQRALDWLAKQPSTTEENYDDDDSSITEAPAKESRPLE<br>AEKKIVKDPKFRNQFRRN-

MmCPR8	MK632956	MYSVKVTVLIALCASAWAAPQRPAAGGPDKDATIVSQQLEVNFDGNYVNNFETSNGISHQESGQSKQVENENPVVSQGSESWSAPDGQQVSLTWTADENGFQAQGSHIPTAPPVPPEIQRALEWNAAHPEQDNEGQGSPARPPPRG-
MmCPR9	MK632957	MYALMIVMAIAPIALSAAAPAEDVIPIVSQTQDGPNPDGSYKWAYESGNGIKASEEGQVANPDSDNAAMQATGEYSYRGDDGQEISVKYTANEEGFKPTGDHLPTAPPVPPLIQKALDWIAAHPSKEDQNNV-
MmCPR10	MK632958	MNSKIILSICLGLVIGISGQRVETRPSSKSSPAVPPSPQASSGSPPKDQATIVRQSQDINPDGSHSSQWETSDGIVVQETGVVKNAGQENETQSVQGSASWTAPDGQKISISWTADENGAVFAGDHLPTPPPTQPIPEIIQRAIDWAAKNPSPDYKKE-
MmCPR11	MK632959	MRFFILLVIVGAAVAQNQYQNQGRQQQQQQYQDDDQQRSQINDRQYSTTTYIPILQWNKEQEHDGTYRTSYETGNNIIAEESGYIRSTGEGEDKVDTLVQSGSYSYTSPEGQLISVTYTADETGFHAIGDHIPTPPPVSEEIQKGLDLIYAGIKKQQEEAAHDQQPPKESQLLSQQRLDRQEDNKKYRF-
MmCPR12	MK632960	MKVLFAFLGLATIVCAQLKYPQYNPDYYGRRYAILRQNHDQNIDGSYAYSYDTENGISVAEQGSRKNLGVGGEAEVVRGQYSYTAPDGTPILVTYVADENGFQAAGAHLPTPPPIPIAIQRALAHNAAHPEEEEPYNRRYYGQKK-
MmCPR13	MK632961	MKTIIILVVLASVASIGLSAPQQEITIIRQEENNSIGVGGYHFSYEQSDGQKREETAELENEGTDDEAMKVVGSYSFVAPDGKTYRVDYTADREGYHPVITLV-
MmCPR14	MK632962	MKFLVVAFALVAVAAAQHQHVTPVPIAILRQSQDQSPDGAYQYSYDTDNGIHVEEQGQPGPVNEEGTPAVVARGAFSYTGPDGTPYQVTYTADETGFHPEAAHLPVAPPVPEEILRAIAYNAAHPEENDGTVSGSFQPGSSVRTAPTPQGSQGFAKPNFGRRFIRYSSDELAKCE-

MmCPR15	MK632963	MYSIIILGLIGSSIAQDLRQPSILSEARYLSGDGTFGAAYTQDDGVQFKEESDANGDRKGSYSYIDPTGQRRTVYYTAGKNGFQASGDGIPEITPPTPEYEPLPEYNPPDYRPPPPRSYKFQTDPDARQLTYDSPSPSPPPPPRSRLHSHPHQLPARPRIIYQPQYQPEYSPVTQPPEVHEYQYEPRPTPTYKPRLQIRYTPAQPQPQPQPQPRFQSQPQLHYHQPKPQYQPEPEYNEPVPQYQQTEPQYQRPDTYRPYVEYSFQTPTNPAPRPRYNEITTPAPRRFYPPGKLDFTRTSDGFSYTFNKN-
MmCPR16	MK632964	MENLKIAKGSTLVLCMLIGSLVCSADKLSPGQKAPPHATNSGGSSLKAPFAGNRAADVGSRSDVGRSGGSRPPATPPQAPKEDGVTREETEDLKEIDGQLVRVVKGNFGYDSPEGLPVAVKYEADENGNRASFTIGNPAGGGAGRGTGTGGRGGGTGGKGTGTGGKGGGTGGKGTGAGGKGGGTGGGSGSGGGKPNSDKTYLPPS-
MmCPR17	MK632965	MTSLFQLTSKILLALCCVRTATVFAGLLPEAGSNGYQYNRPITTTGGGAFAGLGGIVDPSNRRSGYTRDSSSGRLPLGLNPLTSPGFGGSGSGSGSVSGSGSGSVVGGGSVNAFDFGGRTTNTYSAPGFANDFARGIGYNPRPTNTYNLPNGYNSIGNYQGDGSIAPGYNGINDPYRNRYDLDDNRPRPYSFQYQVFDPPSGNDYGQQESSDGNVVQGEYRVLLPDSRTQIVKYTADNVNGYNADVQYEGQAQYPNTITANSPTSVGYQFPGSASEGPYQPTGFPGLIPYQGKRGGVGGISGVGGYSRPNGDRFTGANNNIFGNSGGVAVSTGPGNQYLPPTGGYGK-
MmCPR18	MK632966	MVSIKLLASVMLVVAVAIERSTSAPHKAHSFQHFHGPVEGEGVEVVWKDKHGHEHVDYKAHPKYKFSYGVEDHHTKDFHGQKEHRDGKDVAGEYTVHEPGGNVRTVKYHADHHGGFHAIVHNSGGNDHSGGTYGHGHS-
MmCPR19	MK632967	MPSGLTGILILSAICVISVRANGHAHSFQHFHGPVIGDDQEINWVDKHGHHHHDYVAHPHYEFAYGVKDQHSKDFHGQKEHRDGKEVTGEYTVQEPGGNFRTVKYHADDTGFHADVHNSNGNDHGDERRR-

MmCPR20	MK632968	MACKLIVILAVVLTANAAVVPVAPAAQLLQPASVAVPARIEDYDAVPQ<br>YSFAYDVQDALTGDSKAQYETRNGDIVQGSYSLIEADGTRRIVDYTAD<br>PVNGFNAVVSREPATFAVPAAPAVEIAPAVPVTPVAAPVAPVAPFGFGP<br>GQVIPGGPGPAPAPAVPESGPDSDVEVIDARSGPLRAQAREQSQNDVES<br>GQQRFSSRRIASADARQNSAPLRAQATRFSPEEQRAAPETNARNAAPAP<br>APAPGAPVQARAQVAALTYPAYSAYAGAYSSPFAFTAPVAGLTYTNLN<br>F-
MmCPR21	MK632969	MLALTALTVLLATAVTARPEPPVLGGQQYLPPDQQYGPPRRNNGFGTG<br>GISGQSGSNGGGFGNNQYLPPNQQYGAPGGNNGAGYNDGYNDTPAKY<br>EFEYMVNDIESGNDFGHKESRDGDVTRGTYYVLLPDGRRQTVEYIADQ<br>NGYRPVVTYMQEGNGAGNGYRNDGAGNGYPGGNNGYRY-
MmCPR22	MK632970	MAYKVFLFAAVIAVGSVKAAVVPVAPAAPVAPVLAKLEDFDAVPQYS<br>FAYDVQDALTGDSKAQYETRNGDVVQGSYSLIEADGTRRIVDYTADPV<br>NGFNAVVNREPAVAVAPVVAKTAIPAAPVAAKVVAPVAPVAAPVVAS<br>PYAAPVAAPVVASPYAAPVAAPFVATQYAAPIIARNFLPEISPLITARAS<br>PLVSAPIVVRQPQIIKPARIVGSPAVKQTSRPAKFSKANNNKVAQLSAPL<br>TSAAAGYVY-
MmCPR23	MK632971	MAYKVFLFAAVIAVGSVKAAVVPVAPAAPVAPVLAKLEDFDAVPQYS<br>FAYDVQDALTGDSKAQYETRNGDVVQGSYSLIEADGTRRIVDYTADPV<br>NGFNAVVNREPAVAVAPVVAKTAIPAAPVSAPIVASPYAAPVAAPFVA<br>TQYAAPIIARNFLPEISPLITARASPLVSAPIVVRQPQIIKPARIVGSPAVKQ<br>TSRPAKFSKANNNKVAQLSAPLTSAAAGYVY-
MmCPR24	MK632972	MYALKFLVLIVAILMIKTKASPVEYENYDYDSYNQEPESSYEESYPDHG<br>YSGYEYDSHDNYAFNYGVNDPYTGDVKSQEEVRVGDVVKGSYSINEP<br>DGTIRVVEYTADDHNGFNAVVKKIGHSVHPIPPIARYHPIVPTQYPFSHQ<br>QYLY-
MmCPR25	MK632973	MFKIFVLAALAVASEASYADYDGYVNSHVGYGSYYGLGHGHDDSYET<br>LVAPVVPNAHLGVYGHGYEGHDYDGHNAYAHGLNHHDTYAPAKYAF<br>NYGVNDPHTGDVKSQEEVREGDVVKGSYRLNEPDGTIRVVEYTADPH<br>NGFNAVVKKIGHAVHPSPVPIVKYVAPAYHHGY-

MmCPR26	MK632974	MIKILSLFLTSGICNAVLIGHNGAHGNFYPDYSSYEGVSNDYEGHHVLP<br>TVSVPIHSVSAAPFHDVAHHDHQPHHDFHQQHDLHQAHHELHQAHH<br>ELHQAHHEHHQLEPHHVPIEHHDHGEDHDYYSHPKYTFNYGVHDPHT<br>GDVKTQHEIRDGDVVHGSYSVNEPDGSVRIVEYTADDHNGFNAVVKK<br>VEPSLHPAPHHHTVAHHPGPAPVHVGKFVSAAAPDYYGYEFDKHHLA-
MmCPR27	MK632975	MIKILSLFLTSGICNAVLIGHNGAHGNFYPDYSSYEGVSNDYEGHHVLP<br>TVSVPIHSVSAAPFHDVAHHDHQPHHDFHQQHDLHQAHHELHQAHH<br>EHHQLEPHHVPIEHHDHGEDHDYYSHPKYTFNYGVHDPHTGDVKTQH<br>EIRDGDVVHGSYSVNEPDGSVRIVEYTADDHNGFNAVVKKVEPSLHPA<br>PHHHTVAHHPGPAPVHVGKFVSAAAPDYYGYEFDKHHLA-
MmCPR28	MK632976	MAFKFITLLALVAAANAGFIPAHQPLAYAAAPAYATPVLAKAIHAEDD<br>AHPQYNFNYDVQDSTTGDFKSQHETRDGDSVHGSYSLLESDGTRRTV<br>DYTADAINGFNAVVRKEPAHVAVKAAPLAYAAPAIAKVAAPVA
MmCPR29	MK632977	MAFKFITLLALVAAANAGFIPAHQPLAYAAAPAYATPVLAKAINSEDDP<br>HPQYSFSYDVQDSTTGDFKNQYETRDGDVVQGSYSLLESDGTRRTVD<br>YTADDVNGFNAVVRKEPAQVAVKAVAPAALAVKAAPIAYAAPAAYA<br>KVAAP
MmCPR30	MK632978	MCVKNMNTVVRQITLTLMVSSLGIVYCEPPLSNQYGVPANLPVSNNHE<br>SQGGITNSYGAPLGPSPDGHTHHDEHQDYIDSQPKSYEFGYAVKDSAT<br>GNDFGRQETSDGETVRGEYRVQLPDGRIQIVTYTADWRTGFHADVRYE<br>GEATYPDQYNTGYNNNNNNNNINNKNNKKNNNKN
MmCPR31	MK632979	MATKFLVTLGSILAVAQAGVISGPVAVPVAKAVTDYDPHPQYSYAYDV<br>QDAITGDSKSQHETRNGDVVSGSYSLIEADGTRRVVEYTADPVNGFNA<br>VVHREPAVVNAVPAPAPPPAPIAPAAPTFIKSAPVPVAPAVPVAPALPAP<br>HAFPASYSPYSPFSSLAPFPSPSGGFPAYARSAFFGPAPVHAPAPVYPFR<br>F-

MmCPR32	MK632980	MWRIKIIVLCLTVINVLGDLPPGTRRNTYLPPEPTKGYNYNTPSVPFPRP<br>TPGFPSSRPTSPFPVGPTRPTPTFPTGPSRPPTQPGRPGGGYPTPGPRPTP<br>GFPTPTNRPTGGYPGPGPRPTPGFPDYPGQPSGNNGNTVPGHEHHHEPG<br>MPFDFNYAVKEDAFGNDYSHNAISDGDIVKGEYRVQLPDGRTQIVRYT<br>ADWKHGFSAQVTYEGTPRLDLQRPPGAFRGY-
MmCPR33	MK632981	MTMELVLLVAVSVLVGFQEVTAGHAHSFAHFHGPVEGPGHEVTVHDK<br>HGHHHVDYVAHPKYKFAYGVQDHHTGDYHGHKEHRDGKHIVGEYTI<br>KEPGGNVRTVKYYVDPHGGFFADVHNSNGNDHSGGTYGGYHDHHDY-
MmCPR34	MK632982	MISKALIVVGTMLVPIIFGVPQGPSYLPPSGGRPTGGGTGHPDDWAGDP<br>ANYEYSYEVQDAGLGVDFGHREMRKDEEAKGSYHVLLPDGRTQFVEY<br>VADAGGYRPVIRYEGTATYPASGPSGNDEGYKY-
MmCPR35	MK632983	MAQRFVIIASFLALARAAIISTPSAATVAVSRPTIVGTEPFDHHPQYSYSY<br>AVADDYTDNKAQQETRNGDIVQGHYSLVEPDGSRRTVSYAADGVNGF<br>NAVVQKNHNINVNSQHTVASSVTPVRNAVVSGPGHIWTRAYPNGPSA<br>VVATANANVWRHSSLGTSPYVTSNLVEVGARAGALYDTQPWVGSLG<br>YDSARVVRIH-
MmCPR36	MK632984	MSMIHILSFIGWLLMGIELSITHRYNWPHDAQSMYKILKPASYRVPKYP<br>SIIYVTTPQRKESDDDYMDYPMYSFNYGVADGHTGDSKSAWEERNGD<br>AVRGEYSLTEADGSIRTVTYTADDKNGFNAVVTKTLPQNPY-
MmCPR37	MK632985	HQQHQHQHQDYQHEHVPVATSYMHFHGPLEGPEFQVKVPYVIPHHYE<br>NHYQHSEHQHQENNDLAGHHSHVDEHHADEAHGYIGHTTDYVAHPK<br>YEFAYGVEDHHTGDFHEQREVRDGTSVSGEYRVKEPGGSVRVVKYKA<br>DKDGFHAHVETKGKNDHSGATYTTHAHLHQHEPTHHDDNQNHHHLQ<br>LHAEDTHYEYSHDAYA-
MmCPR38	MK632986	IKFFQVIIILSIATSVTLGSLEPDGYKPPHSAGQSDHYLPASLSSSSHRSILP<br>NQGLTPESHYLPTSYQSPSQNNNQYTGGAVGAGGASGAGTGVVGVVG<br>VVGASSAGGQPRQSNILASGNINQPPGNYYPSSPIGYPDESFAGYDIHQP<br>LAKYEFEYRVNDEYGNDYSHKESRNGDDSTQGVYTVLLPDGRKQIVH<br>YTADQDGYKPKITYEESPTGYGQGGYQY-

MmCPR39	MK632987	MSLIILSVLFLSISFCCGENYQFASEYKIETTTLPPPPVPYSFNYKAGRYPG<br>HVDRYRSESGDGAGVVHGTHSYIDPKFKIRTIHYTADKNGFHPILKNFE<br>DVQAQPQDSEAVRLEKEKHHRLYEKIANSNANPDNLISNLPRDTASVA<br>RAKNHHFELFKKIAAEHEAIAQQREAERLAFEATSVPNNVEESINYYK-
MmCPR40	MK632988	MFRLSFLVSFVSFTTCTPLWPGLQFHNQDGSGSYSYGYQGPHHAKMES<br>RSNGITQGGYSYIDANGILQNVAYTADPVNGFRVKASNLPQQPDNSLLP<br>VNETPEVAEARRNHLAEYTRIANSLGIPQTSNTIDQFEQSTPKNPFILSNE<br>EASRIEIPQPTLNQIYIIKRPSQSLLSHYNGFSSPSAASPVPVPPEAIRASSA<br>VPSNSLVIPVSYRMLHSSLRHTQDSLGQYDYSYTGDTSAKTESRSLDGT<br>TRGAYSYIDANGLLQQVHYIADHNGFRVLATNLPEA-
MmCPAP1-N	MK632989	MIRKVFIFCVLCVQVFGQKFSRRPIPIFRDQMPELLLPNNATAIRENIVDT<br>FSCENRIYGYYADIDNECQVFHVCMPQNRGSRRWSFICPAETVFNQETF<br>VCTRINESIPCEESEQYYVLNEEFGKEEPEDTENEEDNKIDGENQPTNPQ<br>DNQQAPGYAPDTGYTNEVPAVTPFSRNPRTRARPGRRGKNSRFGTTLP<br>GKNERYDTTPLNFIQ-
MmCPAP1-H	MK632990	MRRTCLLLTLYGFLLASAVSAGLKNSRPKFKIATTSTTESPSLDDESEID<br>GNKTEGLDGNSTASHNLTGIPQIDYIWDPNLPRELNGYNLSDYPFYETIP<br>KDIDFKCDGLHDGFYASVPHKCQVYHHCLYGTRYDFLCANFTAFDQK<br>TFICHFVSEIDCANSKKYWHRNDALYQAASTTTIAPPPTTTTTT-

MmCPAP1-K1	MK632991	MKFTAYLLLLMIGCTWRETISTCVLESDFGFMLNCVFKKSSLFRVRNLG<br>GVKGYVGLGFSVGDELGFRESLSNLDISKRRSVQTDAGNNVGIGNGFIK<br>RDDGRANAKIVPPFKPLPPGASRPMAPQPEHQRLVVQKNSTALRTIPAP<br>SLTAMQEAAALRQQKLQQQQKLALQQQHQQQQLQQKQLQQQQLQQQ<br>QQLQQQQLQQQQQQRAQYEAKLRQQQRLQQEAVALKVLKEQRQQQQ<br>EALAQYQKLSLQHQQVMPQSLGLQTKRIQVPQTIVVNSNDVSYSQPLP<br>HIVAAIPPAGSVVNMNNRIATDSKPLTSVVSVSNSGLPFIAMPESSAQLT<br>DRISHSPAVSQESDNQLSKDNYIQLPLPNDEMIASVNEMTLQSLNPAST<br>AEALESSKQQNEKRQSLPSLAPIQGMETSLKALAEASNITLEALEAAILL<br>RQQQLLDKQQGTTTTSSTTTQAPIKTSYTNSGTTKVMNAPREYYPMSY<br>DKNFDDNFASRVDLPDTSFYCGDQKHFPGLYADEDLGCMVFHVCALT<br>DQGLIMKSFLCPESTLFDQTILKCNWWFYVDCKSSKSLYDSNIPISKSYQ<br>LMKALAFFSAYKNHDNSTANSESGS-
MmCPAP1-B2	MK632992	MMIKNFLITFLIFGFFFHWTKGLNVTADADIDDDEDEIDELNFEEFKKSIR<br>RYRRLIPYMSYYVANNYVNQNGQFESSSQRQPQRQRTETVRKPTRINT<br>KDYQTKFIPSVQYDPKEIGGDNNYFMPVRYNTKINYDEINYQEYNKRR<br>PYMEITTPSTVTETRYYKERPRVPYNPVPLGQSSQLKQTRRPDSYLFDY<br>VRPKPTPVNSEYYPVDYEPIRYIKVEPSYQPALAPAPAPSVPVHHQQQQ<br>QHHHQHQQQQSSSRVPLKVPANRPNYISRNSGELNEENLIKSLQLTNQL<br>PEMLNRDNIDSSIKTLVEILTLLHGAKRQHNIGDNSLSAQNIPVAGLGPG<br>PGPSPGTVPVSLPVPGVSGQIYEEYKQQKHQYSRPKVVTEMRYQATSP<br>APAPIPIPNPIAAESLRFTAAGAATGSTVSGIKPSALYGRNHVKPQPSEYS<br>DYNSGETQLRNQNFVEYYTPLIQDIDEKGNRGYLPNKFKPKHKESTYEI<br>AEEVDEVGQEINTPLMDGYLDGIVVTDSKNSGTKGTPGVDYPTYTDIPV<br>TDFSCKDQRYKGFFGDPATRCQVWHYCDLNGGKSSFLCPNGTIFSQIAL<br>TCDWWFNVKCESTTQLYVLNERLYKFILPIMPKFPEDFSGPEVDRYLEL<br>KFKEMEAKLKAKKLKKAMEKKGKDKPQVQDPESEPRAGLGSGSEPGP<br>ELEQI-

MmCPAP1-I	MK632993	MSLKNVFVVVIAGILWSCVLGLQRPAPRYSQQFMPETSFSCRNKIVGSYYADPETDCQLFHVCVSVAGLIQDYKFLCPNDTAFDQESQTCADWYDVDCEAATLYYASDNFDLYRLGSGLESLHYDSIRTDAEPQDHLQRSETNDPVRSAANNLNRVAQNTYTSQKSAPASSTNSNSNNNNNNNNNSN
MmCPAP1-1	MK632994	MIRLVFFVTVLIICQNTNGLDPKIEEEAPKKNHAHRRAVDGSRGNTYYEPTVDDSFDIYDPFDEETDLSSYRLNVPGEPGVDYPSYRRIPQTSFTCERQFRGYYADEEAGCQLFHVCDGNFLVSSFLCPIGSTFSQRYLTCDWWNKVDCSSTKSFYQIETSSPTSEVIDDDDEYLRKAYEMTSLQSSGNGVSVDPQDAQSGDLINETPNANDLRSSERFSDYSNDQSGLDYSQYKYSSRYSNSGSGNQRNRNSRYYSQSSNNNDNNN
MmCPAP1-M1	MK632995	MNLALHRLLLQVILLILQLHHGFRLCSAGEPGYLDFDNLPETNFSCQGKVIGGYYADVEAGCQMFHVCTIGQKDEIMDIKFLCLNGTVFDQETRVCERVDEVDCSKSERFYNLNLELYGNNAVTLSLHEGEDENEETPVVIEDHQVGVTASSQSSTSTTSTTTQRALISSTTAASGSYQHPSGYPQHYQPQPPFPSLQTSQSKSLYDDKNGGYHRQYIYHIGNDPNVNVNNNNNYHNNHNNNNNNN
MmCPAP1-M2	MK632996	MNLALHRLLLQVILLILQLHHGFRLCSAGEPGYLDFDNLPETNFSCQGKVIGGYYADVEAGCQMFHVCTIGQKDVVTDEIMDIKFLCLNGTVFDQETRVCERVDEVDCSKSERFYNLNLELYGNNAVTLSLHEGEDENEETPVVIEDHQVGVTASSQSSTSTTSTTTQRALISSTTAASGSYQHPSGYPQHYQPQPPFPSLQTSQSKSLYDDKNGGYHRQYIYHIGNDPNVNVNNNNNNYHNNHNNNNNNN

MmCPAP1-K2	MK632997	MKFTAYLLLLMIGFSVGDELGFRESLSNLDISKRRSVQTDAGNNVGIGN<br>GFIKRDDGRANAKIVPPFKPLPPGASRPMAPQPEHQRLVVQKNSTALRT<br>IPAPSLTAMQEAAALRQQKLQQQKLALQQHQQQLQQKQLQQQL<br>QQQQQLQQQQLQQQQQRAQYEAKLRQQRLQQEAVALKVLKEQRQ<br>QQQEALAQYQKLSLQHQQVMPQSLGLQTKRIQVPQTIVVNSNDVSYSQ<br>PLPHIVAAIPPAGSVVNMNNRIATDSKPLTSVVSVSNSGLPFIAMPESSA<br>QLTDRISHSPAVSQESDNQLSKDNYIQLPLPNDEMIASVNEMTLQSLNP<br>ASTAEALESSKQQNEKRQSLPSLAPIQGMETSLKALAEASNITLEALEAA<br>ILLRQQQLLDKQQGTTTTSSTTTQAPIKTSYTNSGTTKVMNAPREYYPM<br>SYDKNFDDNFASRVDLPDTSFYCGDQKHFPGLYADEDLGCMVFHVCA<br>LTDQGLIMKSFLCPESTLFDQTILKCNWWFYVDCKSSKSLYDSNIPISKS<br>YQLMKALAFFSAYKNHDNSTANSESGS-
MmCPAP1-B1	MK632998	MINWRSVFIVACVEALVLFAYSVNADGSSEIYNGTRSSNNNNHNNNNN<br>NNKVSKRAKPLDPEDDYYYYDDVDERNNPTNDSPVVPPGFLSPSVREY<br>LDLGKSIPGRPGTDFPVLGKVPYTNFYCDDQEYPGFFADVETRCQAWH<br>YCDIDGRQATFLCPNGTQFSQAVLVCDWWFNVRCELSPKLYAINARLY<br>QKPTESPTRPHRLITKELLENIFARRK-
MmCPAP1-F	MK632999	MISKFICCLVLVAGAILYRSTTAQIDGYRPGVDYPVYNSVPSGLQFNCG<br>GKLPGYYADPEARCQVWHWCLPSGQMFSFLCPNGTVFSQTARVCDW<br>WFKVDCNDSPRLYGINDDLYRDANGNRI-
MmCPAP1-G	MK633000	MHRVFLLMWVAITAAQNPYNLDTRSVSFPGKPTYGPLYSPGIGSSSGG<br>YRDDSYEDNVVTPTPSPTPLYRPAQQPSYRKPNQGSISVLSGPRQGPVR<br>GASYAVQNPQIEEEPLEEEKDEPDRLSLLLPQSKFDCVNKQTGYYADEE<br>LNCEVFHYCQDNAKHSWICPEGFTFHQVHLICMPPNGDISCKKSSQYHF<br>VNEYLYKPLNLQEAESKPNVTLRYSERYFPGDIFTDEREGEEYQQTPSP<br>RRTLHNSQLYLNQPSQTTLNSIPAIARPTPTPQGIPQYRLPVNQVFRSPEE<br>VNIPLQQRRPQQQQQQQVLRRFPQVRPDEEDYEK-

MmCPAP3-C	MK633001	MKYYLISAILILGSNAQETFKCPDDFGFYPHHLSCDKYWKCDNNVAEL<br>KTCGNGLAFDASDAKFLTENCDYLHNVECGERTQLEPPISTPHCSRLYG<br>IFPDEKKCDVFWNCWNGEASRYQCSPGLAYDREARVCMWADQVAEC<br>RIDEVAGGFGCPAAGEIAGASGSFSRHAHPEDCRKYYICIEGIAREYGCP<br>VGTVFKIGDSDGSGACEDPEDVPGCEDYYGDLDLKSIRKSELLAGTDQS<br>NSGKPQAKPRPNHVSSRPQPVQE-
MmCPAP3-D2	MK633002	MRFHPSIIFFVLLIFTQGLAQKEQQEDPCQTKARVVGDIDYCDRYWECI<br>NGRPELFDCPNGLVFAGKHRGVTEGCDYPWRANYCDGKRQANPPIGA<br>EHCDWLYGIFGHETSCTRYWTCWNGTSTEQLCIGGLLYNERSRSCDWP<br>ENVDGCQKHPLCNDDANGNVPLGKSCNRYWQCQGGYPRLQRCPAML<br>VFDRRSLRCVVPPTEDCDVPTTPPTLDGELPDDRNEQEPEEENLPPGVPP<br>LPSGAVPIPLRARPRN-
MmCPAP3-B	MK633003	MRTKIYCLLMVYFGVTTALSRKQQHLELGLRRTAPQGLKEVISPYEND<br>QVNDDEILEVPAQCLEADGYYPDSKQCDKYYDCKDGKYTEKLCPDGL<br>VFNDFSPEHEKCDLPFGVDCSKRPNRQTPQSSQHCPRMHGYFAHENAR<br>ICDTFYYCVEGKFNMITCPDGLVFSEKSGICNWPDEAQKHGCGSRELFN<br>FTCPQVDTAIAATHPRYPDLEDCQFFYVCINGETPRRSGCKLGQAFDER<br>TKKCDWARNVPECKDWYKDQLTDEELYALEHPVSRLKVNDTQSKRR<br>GSRP-
MmCPAP3-D1	MK633004	MFKSIFGFFVLATILSIVNCASLYGLPQCPQEYGEQAYPHPEDCASFFLC<br>TNGTLTLEQCENGLLFDGHGAVHHHCNYYWAVDCKGRKADPTPISSP<br>GCEYQFGLYADTDSCSTTYIKCIYGEPHHESCTPGLVWDDKSHTCIWPD<br>QLIPYCNPEAVVGFKCPQKVPKHTAAAKFWPFPRFAVPGDCGRLITCVE<br>GHPRLITCGDGQLFDSVTLSCMDPKEYPLCAKDA
MmCPAP3-A1	MK633005	MIKIFIISLSIFSVCYGAFTCPKKDGQYEDPVQCDKFYQCEDGVAKEKFC<br>PDGLVFDPLNRKINKCDHVFNVDCGDRLELQPPQPTKKCPRKNGFFAH<br>PDPTVCNVFYNCIDGESVEITCTTGLHFDEFTGTCVWPDSAGREGCGAV<br>GKKLKDGFECPKDRQTDTRGQVVDHPKYAHPNDCQKFYVCLNGDTPR<br>EQGCSDGTVYNEEQQRCDAPENVLGCEDWYKNEEKQS-

MmTWDL-1	MK633013	MRGLIIYFMILGFAWAKPAPEPPVDSYFPPSPGHSGGGGYPSGPVDSYG<br>PPQRPSVIHKHVYVHVPPPEAPEYKPPKYLPPAAAPQKHYKIVFIKAPTP<br>PTPTAPVIPVQPQDEQKTLIYVLVKKPEEAPDVVVPTPAPTQPSKPEVYF<br>IRYKTQKEQSGSYGPPAAPQDSYGPPSSGTGGPY-
MmTWDL-2	MK633014	MRVFMIVALAAVAMARPEAGYSYQQPSGSYGAPGGSGGLGGGHGGSS<br>GHGGGLSLGGGSGGGFLSGFGGGSSLGGFGGGSGGFSGGSSGGALIQK<br>HIYVHVPPPEAPEERPFRPQLQTAAPQKHYKIIFIKAPTPPAPTAPVIPAL<br>QQDEQKTLIYVLVKKPEDAPEINLPTAAPTQPSKPEVYFIKYKTQKESGS<br>IGGGSIGGGSGIGGGSIGGGIGGGSIGGGSGIGGGSIGGGSHGGSLGGGSG<br>SIGGGSIGGGIGGGDGHGGTGPSGPSGNYGPPGQSGPY-
MmCPLCP-1	MK633006	MKMKMQLALVAVLAVCQLAQAESKDNKSIQESSADSQTEKKTEKRGL<br>HHSYGDFGGYGGSYGGSWDHPHEEHVKTVTIEKKIPVPYEVTKHVPYL<br>VEKPVPYEVKVGVPQPYTVEKHVPYPVKVFVKVPVHVPQPYTVEKKIP<br>YEVKVPVDKPYEVKVYVPQPYTVEKHVPVHVKVPVPQPYTVEKHVPY<br>PVKVKIPVPAPYPVEKPVPYEVKVPVDRPYPVHVPKPYPVTVEKPYPVP<br>VDKPVPYEVKVPVDKPYPVPVEKPYPVPYKVHVPQPYTVHKHVPVPV<br>EKPVPYPVKVPVDKPYFVEKHVPVPVEKEVPVPVKVPVPVPVHVHHQ<br>HHTHHDHHTHHEHTHSHSHEGGFEPSSGYGGGDEGGSYGEHSYH-
MmCPLCP-2	MK633007	MIFIVWIGTLALITIARASDYHESHTYEEKIKPVEVPVYKKYAIPIPHPVA<br>VKVPQEIRIPIPQPYHVPIQIPQPYPVEVVKHVDVPVEKHEPYVVEKKVP<br>FVVEKPYPVYVDKKYPITVNKPYPVYVPIYKHVFHHKH-
MmCPLCP-3	MK633008	MRVSLALLALLLTSSVSCKEDKEKDSEAAESKSVESTDSAVEKKQEKR<br>GLYSSEGNYGGGYEGLFMSTGHDSYEGGSHGHSFESHSYPHHEEAKV<br>KEITIVKHVKVPYPVEKKVHYPVEKEVPYPVKVPVAEPYPVEKKIPVPY<br>KVYVKVPVHIPAPYPVEKKVHYPVHVPVERPVPYKTYVPAPYPVEKKV<br>HYPVKVPVPQPYPVEKNIPYPVKVPVHVPQPYPVEKPVPYPVKVHVDR<br>PYPVHIIKPYPVHVEKKVPYP

MmCPLCP-4	MK633009	MFLLIFLVTNVLAHESDNLQWQHHGNYVRNIESNNLPEDSRQNFNYNH<br>NDNGNNFYAGQVNNEFNDQSLRQSSPGNWNFNHNHQSQGPVTINEHV<br>EITKPLALPVFKYIGVPYQQPVEIQVPHTVAVRVPQPYPVHVPVMQPVG<br>YPVMKTIFVPVEKKVPYEVERIVPVPVEQPVPIAIEKPIPVPVDAPYPIHIP<br>IYKNIYHRKVKGRGPRGRERRYKG-
MmCPLCP-5	MK633010	MGHVIHVTLTLIIYCVAVVVSATIRYNYKKPILDCGYLRITPEEKYSWER<br>PNFYVNYRTIDPDDIRDPSIGVLKKEDSKNYISKKQSMDLSVLTDRMSE<br>DTDSTGTLSDQPLIRPTDNIAQSSDSEPRFYDEDQKFTEEYSEHEKVITV<br>VKNVAVPYPVEKQIPYPVIKKVPYPIHTPVAQPYPVEKEIPYPVKVVIKV<br>PTKVPYPVPVYKEVPYAVHVPVERPVPYRTYIPDPYPVEKKVYYESKIP<br>VPEPYPIEKTIAVPVKIPVSVPQPFPVEKPVHYPVEVKVDRPVAVPVEKP<br>YPVTVFKHVPYPVFKPKPVPVEVKVERPVPYPVVKHVPYPVKVEVPQY<br>YPVEKEVPYPVVNHRPYPVPVSVERPVPVPVEKPVPVPVEHEQVEHEP<br>EHGYDDVVKSEIYSGYKHESCCGDEFRPSAIDTHETEYKYGDRNDWD<br>HDKLIHSDEVVANAGVKAETQDSDVRPVVEDSKEDDTNSAEVRNNVA<br>ANASADGAKEATDLKEVNSEGVVVKNDNETINEIDNNNNNI
MmCPLCP-6	MK633011	MKTIVLAAFVAITSAFEKESYETESKEPVINKFDLPISYPVFVRVPEHIGV<br>PIPQPYPVQLAIPHPVPFEIVKHVEIAVEKPEPVVVEKQVPFIVEKPYAVT<br>IEKRFPVTIPKPYPVHVPVYKYVFHHQFKSKGRGH
MmCPLCP-7	MK633012	MKYLLVFALLATAALAEEKKVEEKSADDSSSTSTKKNEKRGILGLGYG<br>YGYDVGGYEIGAGHGHGHGGHGDNGFHNLHDFEFDNLDSYGGGYHG<br>DHHYKTVTVVKNVPVPYPVEKHVPYPVEKHVPYPVKVPVPQPYPVEK<br>HVPYPVKVYVKVPVHVPQPYPVEKKVHVPVHVPVDRPYPVKVLVPQP<br>YPVEKHIPYPVKVPVPQPYPVEKKVHVPVHVPVHVPQPYPVEKIVHVP<br>YKVHVDRPYPVPVPKPYPVHVEKKVPYPVDRPVPYPVKVPVDRPVPYP<br>VEKPVPYPVKVHVPAPYPVEKHIPYPVEKPVPYAVKVPVDRPYPVHIEK<br>HVPYPVEKPVPYPVKVPYPVHVHHDHH<br>EHHTHHEHHGYEYPDHGGFEHDYHH

MmCPF-1	MK633015	MAFKLIIICAFLAGAQAHSVSTTSDNIYRSNGNLAQVSTESKTVETPYSS<br>SSKSDVRVSNPSVYTSATPAPQVQVSPATYSKTLLHPYPQAQLNYPTHQ<br>YHQSYAAPTYYSAPVAKYEVPVAKYEVPVVKYATPVAKYVSQAYQV<br>PHQIYQASAPSVYSTPSVYHHEPVYAAHQSPAPSVYAAPQYYAQHVAQ<br>PVVKVAYSP-
MmApd-1	MK633016	MKTFIVFACLAVATARAGIAHGGYHGQGYGGHGYGGHGGYGGGYGF<br>AGHGGAGLSGGLAAGAGTAASLQGGASSLGSGGLGASYAAGAAAAA<br>GAAGVQQIVSGGVTGGRSDIGPAEGPKANVGPQSGPSDLVGPQEGAKS<br>VGGPRTGPASLVGPAAGPSTLVGPSQGTATLVGPSQATASLVGPSYGG<br>VAFYAGAGGINGDDGSSGSAAAAAPGAGFGGGFGGFGGPGAIAVIGGG<br>PGIHGAIGGHDAGVAVINGPSGAIHAGLGSHGAIIPAPIHGHGKWA-
MmApd-2	MK633017	MKCLILCALMATAVSAAPSGLLGHGWVGHGLAGPVLAGSALAGPHVG<br>PSSLSGPVAGPAHISGAVDGGAVVTGSVAGPSAVIGSSAGPAFVSEPAH<br>GLSWGLGHAVHTHDAGAIIVGGHVGHAALHGGLGSVVVAGPDGASIS<br>THGVAPGAIIATGAHHGHLGHWW-
MmApd-3	MK633018	SDSSKGVITDRLLEILIQSYRAQIIALAIFGVAAAGRDRRGAVWGYGDH<br>GAYGAHGFYGGHGAYGAHGVALAGHSVGPASVVGPHAGASALAGPA<br>IGPSHLAGSVAGPVHVSGAVAGPATVTASVAGPAHVEGYDGHYDGAY<br>GHGFAHGYGGFAHGFGHAGYGYGHGAHGGHGVVVVGPAHHGAVHA<br>GYGSHGAILAGPHAHGAVLSGPHSHGAVISGPHSGTAAVSGPHAGDVV<br>ISGPSGKITAHGAGHGAAIHAGYGHGHHW-
