# Supplementary material for: Identification and temporal expression profiles of cuticular proteins in the endoparasitoid wasp, Microplitis mediator
Source: Insect Sci. 2019 Aug 6;27(5):998–1018. doi: 10.1111/1744-7917.12711 (PMC7497268; doi:10.1111/1744-7917.12711)
Supplement: Supplementary file 6 — Table S4. Accession numbers of cuticular protein (CP) genes, used in phylogenetic analysis and Rebers and Riddiford (RR)‐3 proteins annotation. [file INS-27-998-s006.pdf]

Table S4. Accession numbers of all CP genes, which were used in phylogenetic analysis.

RR-1							
**Name**	**Accession number**	**Name**	**Accession number**	**Name**	**Accession number**	**Name**	**Accession number**
TcCPR4	NP_001139387.1	GB46299-PA	AmCPR18	NP_001161365.1	AmCPR19	NV16252-PA	NvCPR19
AaCP2-like	Aalb_OocyteEmbryoAssembly_023662;Aalb_OocyteEmbryoAssembly_023663;Aalb_OocyteEmbryoAssembly_023664;	GB42612-PA	AmCPR20	NV16250-PA	NvCPR17	NV13164-PA	NvCPR34
BMWCP10	NP_001036894.1	GB52161-PA	AmCPR28	NV16248-PA	NvCPR16	NV13462-PA	NvCPR22
NlugCpr3	MF942777.1	GB46297-PA	AmCPR14	NV13159-PA	NvCPR30	NV16251-PA	NvCPR18
NlugCpr8	MF942781.1	GB47906-PA	AmABD-4X1	NV13161-PA	NvCPR31	NV16253-PA	NvCPR20
NlugCpr10	MF942783.1	GB52203-PA	AmCPR13	NV14650-PA	NvCPR40	NV12578-PA	NvCPR38
NlugCpr36	MF942809.1	GB47903-PA	AmCPR24	NV13163-PA	NvCPR33	NP_001161317	NvCPR57
DmACP65a	AAB88068.1	GB47902-PA	AmSgAbd-1	NV20074-PA	NvCPR54	NP_001161326.1	NvCPR49
DmLCP2	AAB27167.1	GB46298-PA	AmSgAbd-8-like	NV14649-PA	NvCPR39	XP_008214445.1	NvCPR49-X1
GB46312-PA	AmCPR22	GB46310-PA	AmCPR17	NV16249-PA	NvCPR21		
GB46311-PA	AmCPR21	NP_001257753.1	AmCPR12	NV14825-PA	NvCPR53		

RR-2							
**Name**	**Accession number**	**Name**	**Accession number**	**Name**	**Accession number**	**Name**	**Accession number**
TcCPR18	AEG74043.1	GB48823-PA	AmCPR2	XP_001122183.1	AmCPR26	NV17361-PA	NvCPR47
TcACP22	P26968.1	GB48831-PA	AmCPR4	NV13629-PA	NvCPR10	NV20082-PA	NvCPR19
AaCP19b	Aalb_OocyteEmbryoAssembly_000706;	GB48832-PA	AmCPR3	NV13631-PA	NvCPR12	NV15284-PA	NvCPR21
AaCP19c	Aalb_OocyteEmbryoAssembly_013372;Aalb_OocyteEmbryoAssembly_036107;	GB40299-PA	AmCPR5	NV13628-PA	NvCPR9	NV15977-PA	NvCPR45
AaCP19a	Aalb_OocyteEmbryoAssembly_013364;	GB40566-PA	AmCPR6	NV13634-PA	NvCPR15	NV15976-PA	NvCPR44

NlugCpr90	MF942863.1	GB42582-PA	AmCPR10	NV13623-PA	NvCPR4	NV15974-PA	NvCPR43
NlugCpr94	MF942867.1	GB42581-PA	AmCPR11	NV13620-PA	NvCPR1	NV16356-PA	NvCPR23
NlugCpr64	MF942837.1	GB45957-PA	AmCPR15	NV13626-PA	NvCPR7	NV16361-PA	NvCPR28
NlugCpr83	MF942856.1	GB52920-PA	AmCPR16	NV13622-PA	NvCPR3 X1	NV16358-PA	NvCPR25
NlugCpr62	MF942835.1	GB52824-PA	AmCPR23	NV13627-PA	NvCPR8	NV16357-PA	NvCPR24
NlugCpr54	MF942827.1	XP_016769565.2	AmCPR8	NV13633-PA	NvCPR14	NV16359-PA	NvCPR26
NlugCpr47	MF942820.1	XP_006561931.1	AmCPR7	NV13625-PA	NvCPR6	NP_001153407.1	NvCPR13
NlugCpr56	MF942829.1	XP_016769566.1	AmCPR19	NV13624-PA	NvCPR5	NP_001161335.1	NvCPR59
NlugCpr73	MF942846.1	NP_001257746.1	AmCPR1	NV18941-PA	NvCPR48	NP_001166268.1	NvCPR3 X2
NlugCpr61	MF942834.1	XP_016769566.1	AmCPR9	NV13137-PA	NvCPR29		
DmCP51A	AAF58238.1	XP_006557278.1	AmCPR20	NV19175-PA	NvCPR46		
**RR-3**							
**Name**	**Accession number**	**Name**	**Accession number**	**Name**	**Accession number**	**Name**	**Accession number**
Nvit58	XP_001601627	BcNCP14.9	P82119.1	MsCPR146	Msex2.06360	MsCPR148	Msex2.07106
BmCPR149	ACY06906	BcNCP15.0	P82120.1	MsCPR147	Msex2.11251	MsCPR149	Msex2.04414
**CPAP1**							
**Name**	**Accession number**	**Name**	**Accession number**	**Name**	**Accession number**	**Name**	**Accession number**
AgCPAP1-A	XP_321953	DaCPAP1-L	XP_001953230	MsCPAP1-D	Msex2.03859	PhcCPAP1-K	XP_002432786
AgCPAP1-B1	XP_310217	DmCPAP1-A	NP_729504	MsCPAP1-F	Msex2.02135	PhcCPAP1-M	XP_002424534
AgCPAP1-B2	XP_001230737	DmCPAP1-B1	NP_650734	MsCPAP1-G	Msex2.00269	TcCPAP1-A	ACY95466

AgCPAP1-C	XP_310283	DmCPAP1-B2	NP_650731	MsCPAP1-H	Msex2.02134	TcCPAP1-B1	ACY95467
AgCPAP1-H	XP_001237672	DmCPAP1-C	NP_650538	MsCPAP1-I	Msex2.03076	TcCPAP1-B2	XP_971785
AgCPAP1-K	CAD29630	DmCPAP1-E	NP_995986	MsCPAP1-J	Msex2.03236	TcCPAP1-C	ACY95468
AgCPAP1-N	XP_315598	DmCPAP1-F	CBA35308	MsCPAP1-K	Msex2.01703	TcCPAP1-D	ACY95469
AmCPAP1-A	XP_001122348	DmCPAP1-H	NP_648179	MsCPAP1-L	Msex2.09867	TcCPAP1-E	ACY95470
AmCPAP1-B1	XP_003249744	DmCPAP1-I	NP_001262938	MsCPAP1-M	Msex2.03103	TcCPAP1-F	ACY95471
AmCPAP1-E	XP_003250709	DmCPAP1-J	NP_001097699	MsCPAP1-N	Msex2.02137	TcCPAP1-G	ACY95472
AmCPAP1-F	XP_001122842	DmCPAP1-K	AHN57230	MsCPAP1-O	Msex2.08722	TcCPAP1-H	ACY95473
AmCPAP1-I	XP_394746	DmCPAP1-N	NP_001246598	NvCPAP1-A	XP_001603626	TcCPAP1-I	ACZ04319
AmCPAP1-J	XP_395554	DmCPAP1-O	NP_725747	NvCPAP1-J	XP_001604297	TcCPAP1-J	ACY95474
AmCPAP1-K	XP_006571075	MsCPAP1-A	Msex2.13160	PhcCPAP1-A	XP_002432783	TcCPAP1-K	EFA11397
AmCPAP1-M	XP_006568320	MsCPAP1-B1	Msex2.00613	PhcCPAP1-F	XP_002423061	TcCPAP1-L	XP_001809190
CqCPAP1-B1	XP_001862964	MsCPAP1-B2	Msex2.00614	PhcCPAP1-G	XP_002423584	TcCPAP1-M	XP_001807896
CqCPAP1-O	XP_001841755	MsCPAP1-C	Msex2.00381	PhcCPAP1-H	XP_002426626	TcCPAP1-N	EFA06939
**CPAP3**							
**Name**	**Accession number**	**Name**	**Accession number**	**Name**	**Accession number**	**Name**	**Accession number**
TcCPAP3-A1	ACY95475	AgCPAP3-D1	XP_560209	ApCPAP3-B	XP_001947981	MsCPAP3-B	Msex2.08808 +Msex2.15015
TcCPAP3-A2	ACY95476	AgCPAP3-E	XP_310082	ApCPAP3-C	XP_001944961	MsCPAP3-C5a	Msex2.08810_a
TcCPAP3-B	ABL73928	CqCPAP3-A1	XP_001842244	ApCPAP3-D2	XP_001947475	MsCPAP3-C5b	Msex2.08810_b
TcCPAP3-C5a	ABL73929	CqCPAP3-B	XP_001847921	AmCPAP3-A1	XP_001120217	MsCPAP3-D1	Msex2.08807

TcCPAP3-C5b	ABL73930	CqCPAP3-C	XP_001848925	AmCPAP3-B	XP_392261	MsCPAP3-D2	Msex2.04890
TcCPAP3-D1	ACY95477	CqCPAP3-D1	XP_001842247	AmCPAP3-C	XP_001121720	MsCPAP3-E1	Msex2.03293
TcCPAP3-D2	ABL73931	CqCPAP3-D2	XP_001868521	AmCPAP3-D1	XP_393551	MsCPAP3-E2	Msex2.03294
TcCPAP3-E	ACY95478	CqCPAP3-E	XP_001845871	AmCPAP3-E	XP_397120	MsCPAP3-E3	Msex2.03295
AaCPAP3-A1	XP_001662353	DmCPAP3-A1	NP_608378	NvCPAP3-A1	XP_001607914	MsCPAP3-E4	Msex2.14886
AaCPAP3-C	XP_001663971	DmCPAP3-B	NP_609339	NvCPAP3-A2	XP_001608254	PhcCPAP3-C	XP_002425178
AaCPAP3-D1	XP_001660247	DmCPAP3-C	NP_649611	NvCPAP3-B	NP_001136346	PhcCPAP3-D1	XP_002429482
AaCPAP3-D2	XP_001662442	DmCPAP3-D1	NP_523418	NvCPAP3-D1	XP_001608252	PhcCPAP3-D2	XP_002429485
AaCPAP3-E	XP_001662027	DmCPAP3-E1	NP_608957	NvCPAP3-D2	XP_001607911	PhcCPAP3-E	XP_002427031
AgCPAP3-A1	XP_309184	DmCPAP3-E2	NP_723116	NvCPAP3-E	XP_001603894		
AgCPAP3-B	XP_318884	ApCPAP3-A1	NP_001156724	MsCPAP3-A1	Msex2.08805		
AgCPAP3-C	XP_319536	ApCPAP3-A2	XP_001947844	MsCPAP3-A2	Msex2.08806		
